# Multiomic Analysis Provided Insights into the Responses of Carbon Sources by Wood-Rotting Fungi *Daldinia carpinicola*

**DOI:** 10.3390/jof11020115

**Published:** 2025-02-04

**Authors:** Peng Yang, Xingchi Ma, Yu Zhang, Yanan Sun, Hao Yu, Jiandong Han, Meng Ma, Luzhang Wan, Fansheng Cheng

**Affiliations:** 1 Institute of Agricultural Resources and Environment, Shandong Academy of Agricultural Science, Jinan 250100, China; yp15926@163.com (P.Y.); hangjiandong16@163.com (J.H.); 2 Key Laboratory of Wastes Matrix Utilization, Ministry of Agriculture and Rural Affairs, Jinan 250100, China; 3 College of Food Science and Engineering, Qingdao Agricultural University, Qingdao 266109, China; maxingchi0601@163.com (X.M.); z17862173918@outlook.com (Y.Z.); 18351573738@163.com (Y.S.); mamengss@163.com (M.M.); 4 Qingdao Special Food Research Institute, Qingdao 266109, China; 5 College of Life Sciences, Qingdao Agricultural University, Qingdao 266109, China; yuhao@qau.edu.cn; 6 Shandong Province Key Laboratory of Applied Mycology, Qingdao 266109, China

**Keywords:** wood-rotting fungi, CAZyme, carbon source, sucrose, sawdust, secondary metabolites

## Abstract

*Daldinia carpinicola* is a newly identified species of wood-rotting fungi, with substantial aspects of its biology and ecological function yet to be clarified. A Nanopore third-generation sequencer was employed for de novo genome assembly to examine the genetic characteristics. The genome consisted of 35.93 Mb in 46 contigs with a scaffold N50 of 4.384 Mb. Glycoside hydrolases and activities enzymes accounted for a large proportion of the 522 identified carbohydrate-active enzymes (CAZymes), suggesting a strong wood degradation ability. Phylogenetic and comparative analysis revealed a close evolutionary relationship between *D. carpinicola* and *D. bambusicola*. *D. carpinicola* and *Hypoxylon fragiforme* exhibited significant collinear inter-species genome alignment. Based on transcriptome and metabolomic analyses, *D. carpinicola* showed a greater ability to utilize sucrose over sawdust as a carbon source, enhancing its growth by activating glycolysis/gluconeogenesis and the citrate cycle. However, compared with sucrose, sawdust as a carbon source activated *D. carpinicola* amino acid biosynthesis and the production of various secondary metabolites, including diterpenoid, indole alkaloid, folate, porphyrin, and biotin metabolism. The study establishes a theoretical basis for research and applications in biological processes, demonstrating a strategy to modulate the production of secondary metabolites by altering its carbon sources in *D. carpinicola*.

## 1. Introduction

Wood decomposition is essential for terrestrial carbon cycling [1]. The rate of wood-rotting is primarily influenced by fungal and insect activity, as well as environmental factors such as temperature and humidity [2]. Furthermore, wood-rotting fungi are integral in forest ecosystems, contributing to biodiversity, creating habitats, and supplying nutrients for other living organisms [3]. Wood-rotting fungi, primarily classified within *Ascomycetes* or *Basidiomycetes*, serve as essential decomposers in forest ecosystems, representing a significant yet mostly unexplored component of biodiversity [3].

The fungi’s action results in the partial or complete degradation of wood’s key components, which include lignin, cellulose, and hemicellulose. Wood-rotting fungi is classified according to rot type, which includes white-rotting fungi, brown-rotting fungi, and soft-rotting fungi [4]. Both white-rotting *Basidiomycetes* and soft-rotting *Ascomycetes* release laccases, key enzymes for oxidative breakdown, without the presence of lignin peroxidase [5]. Contrarily, brown-rotting fungi cannot degrade lignin; they alter its chemical properties [6]. Wood-rotting fungi secrete various enzymes that facilitate the deconstruction of lignocellulose into simpler sugars. These processes involve carbohydrate biotransformation processes such as synthesis, metabolism, modification, and transport [7]. Certain enzymes, such as laccase and xylanase, have been engineered and extensively utilized for processing paper mill effluents and mitigating soil contamination, demonstrating considerable application potential [8].

Certain fungi serve as notable forest pathogens, while others exhibit valuable medicinal and nutritional properties. Approximately 2000 species of macro fungi are considered safe for consumption and possess significant gastronomic value, consequently serving as a viable food source for humans. Edible mushrooms are rich in carbohydrates, essential amino acids, and various biologically active compounds that promote health [9]. Mushrooms contain a variety of secondary metabolites with beneficial properties, such as antioxidant, antibacterial, antiviral, anticancer, and anti-inflammatory activities. Centuries of traditional Chinese medicine have revered *Ganoderma lucidum* (Lingzhi or Reishi mushrooms) as a medicinal panacea [10]. Identifying novel fungal species that possess diverse biomolecules, remarkable medicinal properties, and attractive nutraceuticals is highly significant for human health and nutrition.

Recent advancements in sequencing technology have yielded extensive whole-genome wood-rotting fungal data, promoting improved research into their substrate decomposition and metabolic characteristics. Genome sequencing of the *Steccherinum ochraceum* white-rotting fungus revealed 12,441 gene models, among which 181 were models of tRNA-coding genes, and 12,260 protein-coding genes annotated with different databases. The study provided crucial insight for investigating the biochemical processes underlying the advanced wood-rotting by *Basidiomycetes* [11].

Fungal secondary metabolites (FSMs) are a diverse collection of low-molecular-weight compounds derived from central metabolic pathways and primary metabolite pools. They play a significant ecological role and offer advantages in the medicinal, pharmaceutical, and agricultural industries [12]. The *Daldinia* genus, part of the *Ascomycota* family, primarily comprises fungi that inhabit wood, seeds, fruits, and angiosperm leaves. These fungi are predominantly found in tropical and subtropical regions [13]. Moreover, they produce significant FSMs, while the metabolites derived from their stromata and cultures serve as valuable taxonomic indicators [14]. Previous studies have isolated a range of bioactive metabolites from *D. eschscholtzii*, including ribonic glycosides, lactones, chromones, polyketides, phenolics, and alkaloids. These metabolites exhibit activities such as antioxidant, antimicrobial, cytotoxic, immunosuppressive, and α-glucosidase inhibition activities [15]. Furthermore, recent research has identified new cellulases, xylanase, and β-glucosidase in *D. caldariorum*, *D. eschscholzii*, and *D. childiae* [13]. Although these studies highlight the considerable industrial potential of the *Daldinia* genus, no research is available on *D. carpinicola* in FSMs aspect.

FSM synthesis is influenced by the type and concentration of the nutrients in the culture medium. As a crucial nutritional element for FSM production, the carbon supply requires extensive investigation for both industrial applications and scientific inquiry [16]. Glucose and similar repressive carbon sources can regulate gene expression and enzyme activity to control the production of certain metabolites, such penicillin production and the expression of the genes involved in its synthesis. This repression is triggered by sugars such as glucose, fructose, galactose, and sucrose, and is modulated by the *CreA* transcription factor [17]. However, the impact of various carbon sources on the transcriptional and metabolic profiles of *Daldinia* genus, particularly regarding the synthesis of secondary metabolites, remains unclear.

Therefore, *D. carpinicola* was subjected to high-quality whole genomic sequencing for the deep investigation of biochemical processes of wood decomposition. Furthermore, transcriptomics and metabolomics were used to analyze the effects of sucrose and chestnut sawdust carbon sources culture on secondary metabolites and other important metabolites of *D. carpinicola*.

## 2. Materials and Methods

### 2.1. Strains and Culture

The *D. carpinicola* strain was isolated from the Taishan Mountains in the Shandong Province, China. The strain was cultivated on Czapek–Dox agar medium (30.0 g sucrose, 3.0 g NaNO_3_, 1.0 g K_2_HPO_4_, 0.5 g MgSO_4_·7H_2_O, 0.5 g KCl, and 0.01 g FeSO_4_·7H_2_O dissolved in distilled water) for 10 d at 150 rpm at 28 °C. The mycelium was harvested and sequenced. To explore the effect of the carbon source on the strain transcriptome and metabolism, the strain was inoculated into a Czapek–Dox liquid medium (cdm group) and a modified Czapek–Dox liquid medium in which the sucrose was replaced with chestnut sawdust (saw group). Both groups were also cultured for 10 d at 150 rpm at 28 °C. The mycelium was harvested and sequenced for transcriptome and metabolism information.

### 2.2. Genome Sequencing

Liquid nitrogen was employed to pulverize 2 g of the mycelium pellets into a fine powder, after which a DNA extraction kit (Beijing Solarbio Science & Technology Co. Ltd., Beijing, China) was used for high-quality genomic DNA extraction. The purity, concentration, and integrity were assessed using a Nanodrop spectrophotometer and agarose gel electrophoresis at a concentration of 0.35% *w*/*v*, while the Blue Pippin system was employed to isolate large DNA fragments. An SQK-LSK109 ligation kit (Oxford Nanopore Technologies, Oxford Science Park, Oxford, UK) was used for library construction, followed by adapter ligation, purification using paramagnetic beads, library quantification, and sequencing using a Nanopore third-generation sequencer.

### 2.3. Genome Assembling

The Burrows-Wheeler Aligner (BWA) 0.7.17 software was employed for sequence alignment, while the Benchmarking Universal Single-Copy Orthologs (BUSCO) v2.0 tool was utilized to evaluate the completeness of the genome assembly [18].

### 2.4. Prediction of the Protein-Coding Genes and Non-Coding RNAs

The de novo prediction was performed according to a previously described method [19]. The Gene Model Mapper v1.3 software was used for predictions based on homologous proteins. The tRNAscan-SE–2.0 software was applied to identify the genomic tRNA, while Infernal 1.1 software was employed for rRNA and additional ncRNA prediction using the Rfam database [20].

### 2.5. Gene Prediction and Gene Cluster Prediction

Gene prediction results were integrated using Evolutionary Gene Prediction Method software of EVidenceModeler (EVM) 2.1.0. An implicit hidden Markov model tailored to specific gene cluster types and antiSMASH 5.2.0 software were employed for the precise detection of the gene clusters responsible for encoding the secondary metabolites across a broad range of known chemical classes [21].

### 2.6. Comparative Genomic Analysis

The pairwise average nucleotide identity (ANI) values between genomes were analyzed using FastANI software 1.34 [11]. Single-copy orthologous genes and phylogenetic trees were analyzed using OrthoFinder v2.5.4 software. The circle map and genomic collinearity was produced using Circos 0.69 and Mauve 2.4.0 software [15].

### 2.7. Functional Genomic Annotation

The predicted gene sequences were BLAST compared with KOG, KEGG, Swiss-Prot, TrEMBL, Nr, and other functional databases to obtain the gene function annotation results. Based on the comparison results of Nr database, Blast2GO was applied to annotate the function of GO database [22]. The Hidden Markov Models (HMMER) 3.4 software was used to annotate Pfam function based on the Pfam database. A hypergeometric test was used to identify the significantly enriched pathways of the above annotations (*p* < 0.05) [14].

The predicted gene sequences were aligned with functional databases using BLAST [22] for gene function annotation. The carbohydrate-active enzymes (CAZyme) were identified via the CAZyme database and dbCAN2 database [23].

### 2.8. Protein Subcellular Localization Analysis

The SignalP 4.0 software was employed to analyze the protein sequences of all the predicted genes, aiming to identify those containing signal peptides [24]. The TransMembrane prediction using HMMER 3.4 software was utilized for transmembrane and secreted protein analysis. The secreted proteins were examined further using EffectorP 3.0 software to predict the fungal effector proteins [25].

### 2.9. Transcriptomic Sequencing and Analysis

RNA-seq sequencing was performed using Illumina NovaSeq6000 sequencing platform in PE150 mode. The software HISAT2 2.2.1 was used to accurately compare Clean Reads with the reference genome to obtain the location information of Reads on the reference genome. Then, StringTie 3.0.0 was used to assemble the above reads, and the transcriptome was reconstructed for subsequent analysis [18].

The PCA analysis, heatmap, and volcano map were performed using the prcomp, pheatmap, and ggplot2 packages of R language, respectively. The sequencing data were unloaded and filtered to obtain clean data, which were compared with the specified reference genome. The StringTie software was employed to assemble the reads and reconstruct the transcriptome for further analysis [26].

The DESeq2 1.46.0 software was used for differential analysis, with criteria set for Fold Change ≥ 2 and FDR < 0.01. The enrichment results were visualized using bubble maps created via ClusterProfiler 4.14.4 software [27].

### 2.10. Metabolomic Sequencing and Analysis

Ultra-high performance liquid chromatography (Waters Acquity I-Class PLUS) (Waters Corporation, Milford, MA, USA), coupled with a high-resolution mass spectrometer (Waters Xevo G2-XS QTOF) (Waters Corporation, Milford, MA, USA) was used for detection.

The MassLynx V4.2 software was used for data collection, followed by peak extraction and alignment using Progenesis QI 2.4. The online Metabolite and Tandem Mass Spectra Database in Progenesis QI, as well as public and custom databases from Beijing Biomarker Technologies Co., LTD., were employed for compound determination, while theoretical fragment identification was also performed [28].

The heatmap and radar chart were created using pheatmap and fmsb packages of R language 4.1.0 software, respectively. The Z-score value is based on the conversion of quantitative values of metabolites and is used to measure the difference deviation of the experimental group from the control group. The Z-score standardization was conducted by taking the mean and standard deviation of each variable and applying the Z-score formula of R language. The clusterProfiler hypergeometric test method was used to enrich and analyze the KEGG annotation results and generate a network diagram [23].

### 2.11. The Methods of Combined Transcriptomic and Metabolomic Analysis

UV scaling was used for transcriptomic and metabolomic data pretreatment. Two-way orthogonal partial least squares (O2PLS) evaluated the intrinsic correlation between the two datasets by integrating the two datasets. Then, the transcriptomic and metabolomic O2PLS models were constructed and the scores of each sample were calculated. The load value of each gene and metabolite was determined to obtain the load map [29]. Finally, the top 15 genes/metabolites with load lengths in the first two dimensions (with the greatest correlation) were selected to draw a histogram.

The R language software and Venn Diagram tools were utilized to identify the pathways associated with the genes in the transcriptomic data, the shared pathways related to the metabolites in the metabolomic data, and to create a Venn diagram for visualization [30].

The R language 4.1.0 NbClust 3.0.1 and ggplot2 3.3.5 software were used to classify the differentially expressed genes (DEGs) and differentially expressed metabolites (DEMs) in each group via K-means, respectively, after which the related plots were created [31].

Weighted Gene Co-expression Network Analysis (WGCNA) is a method for identifying co-expressed gene modules, which can be implemented using the WGCNA package in the R language software. An Ipath analysis map was produced using Ipath 3.0 software [32].

## 3. Results

### 3.1. Genomic Assembly of D. carpinicola

A total of 1183.4 million bp of raw data were obtained via the Nanopore third-generation sequence platform, while the sequencing depth was estimated as 32.93×. The complete genome sequence spanned a total length of 35.93 Mb, with 46 contigs. The scaffold N50 value was 1.323 Mb, with a total GC content of 44.81% (Table 1).

The results revealed that the 350 bp library displayed an 81.71% mapping rate, 78.97% accurate read alignment, and 99.97% coverage. The BUSCO fungi_odb9 database includes 290 conserved core fungal genes. The results indicated that 281 BUSCO genes were fully represented in the assembled genes, resulting in a 96.90% genome integrity score (Table S1). The evaluation showed excellent second-generation sequencing data quality (Table S2).

The predicted fungal repetition sequence was 451,150 bp, with a ratio of 1.26%, while the majority consisted of simple sequence repeats (SSR). These findings suggested that the assembled genome displayed a high level of completeness and accuracy.

### 3.2. D. carpinicola Gene Prediction

The gene prediction characteristics were summarized and presented in Table 2. Of the 10,596 genes integrated, 10,509 (99.17%) were validated by both homological and transcriptomic predictions, indicating a high level of predictive accuracy. The predicted non-coding RNA results comprised rRNA, tRNA, and other types of ncRNAs, with respective counts of 33, 159, and 53.

### 3.3. D. carpinicola Genome Annotation

**Figure 1:**
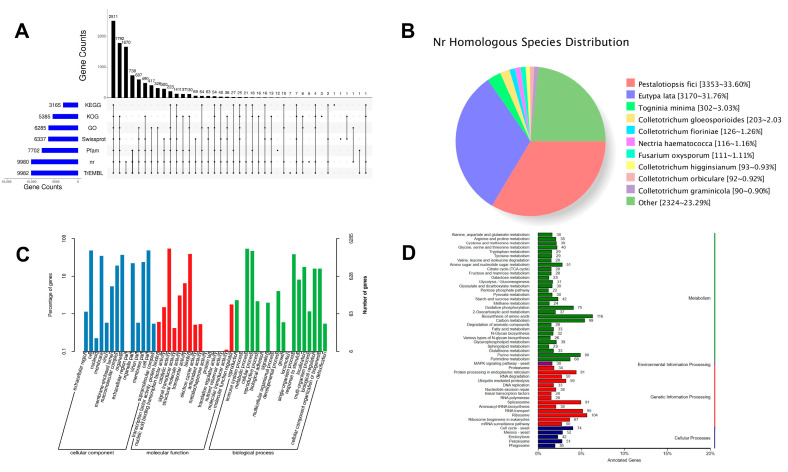
*D. carpinicola* Genome Annotation. (**A**) The UpSet plot illustrating the gene annotation discrepancies across seven commonly utilized databases. (**B**) The distribution map depicting the presence of homologous species in the Nr database. (**C**) The statistical analysis presenting the GO annotations classification. Abscissa represents classification, and ordinate represents the number and percentage of genes. (**D**) The statistical analysis presenting the KEGG annotation classification. Abscissa represents annotated genes, and ordinate represents classification.

The predicted *D. carpinicola* gene sequences were functionally analyzed using the KEGG, KOG, GO, Swiss-Prot, Pfam, Nr, and TrEMBL databases (Figure 1A). The Nr and TrEMBL databases annotated 9980 and 9982 genes, respectively. Figure 1B presents the statistical results of the species distribution derived from the sequence comparisons with the Nr database.

**Figure 2:**
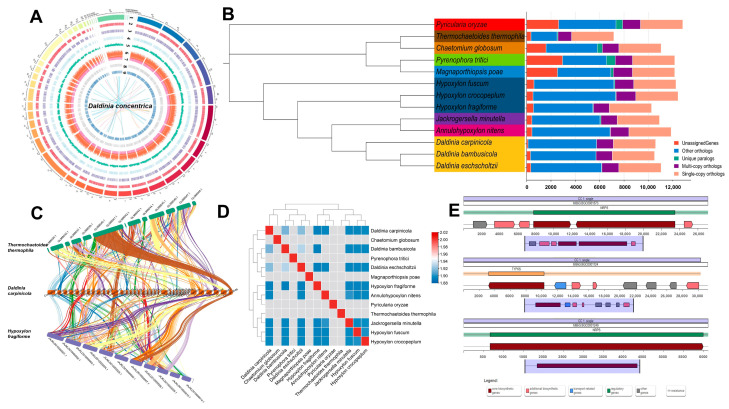
Genome evolution analysis of *D. carpinicola*. (**A**) The circle map of the *D. carpinicola* genome assembly and gene prediction. Nine distinct layers are graphed in an inward progression from the outermost edge. The outermost layer presents a circular depiction of the 46 contigs, with each segment representing 1 Mb in size. Layers two to nine illustrate various genomic features, including the gene location on each contig, the genes on both the forward and reverse strands, the GC content, the gene density, the repeat sequences, the tRNA genes, and the CAZyme genes. The connections drawn within and across the chromosomes highlight collinear gene blocks: terpene (gray), non-ribosomal peptide synthase (NRPS) or NRPS-like (red), type I polyketide synthase (T1PKS) (blue), and indole (yellow). (**B**) The *D. carpinicola* evolution and comparative genomic analysis. Left section: A phylogenetic tree was built using 309 single-copy orthologous *D. carpinicola* genes and 12 representative Ascomycetes. Right section: The number of different orthologous gene types was calculated for each fungal species, with each type represented by a different color. (**C**) The genomic collinearity among *D. carpinicola*, *Thermochaetoides thermophila*, and *H. fragiforme*. Each line connects a pair of collinearity blocks between two genomes. (**D**) A cluster heatmap displaying the ANI values of the 13 strains. (**E**) The *D. carpinicola* gene cluster structure. The gene structures of dihydrolysergic acid, pyranonigrin E, and dimethylcoprogen are shown from top to bottom.

A total of 10 homologous species were identified in the Nr database. *Pestalotiopsis fici* and *Eutypa lata* represented the most abundant species, accounting for 33.60% and 31.76% of the total content, respectively. Figure 1C shows the GO annotation classification statistics. The significantly enriched areas included the extracellular regions, biological adhesion in cellular components, catalytic activity in molecular functions, and biological adhesion in biological processes. Figure 1D shows the KEGG annotation classification statistics. The pathways involved in carbohydrate metabolism included the citrate cycle, fructose and mannose metabolism, and pentose phosphate pathway. Additionally, amino acid metabolism, endocytosis, and phagosome were signally enriched. Protein sequence analysis of the predicted genes identified 1057 proteins with signal peptides. Furthermore, the predictions identified 50 gene clusters associated with secondary metabolites (Figure 2). Circos was used to create a circular map of the complete *D. carpinicola* genomic information according to the genome assembly and functional annotation results (Figure 2A).

### 3.4. Genome Evolution Analyze

Phylogenetic analyses were performed to elucidate the evolutionary relationship between *D. carpinicola* and 12 representative Ascomycetes. The NCBI Accession Number of the strains is presented in Table S3. The *D. carpinicola* formed a cohesive cluster within a branch alongside *D. bambusicola* and *D. eschscholtzii*, suggesting a close phylogenetic affinity (Figure 2B). Therefore, *D. carpinicola* was cultivated and applied in the same way as *D. bambusicola* [29,30] and *D. eschscholtzii* [31,32]. Furthermore, the findings revealed that *D. carpinicola* displayed a pronounced evolutionary relationship with *Hypoxylon fuscum*, *Hypoxylon crocopeplum*, *Jackrogersella minutella*, and *D. bambusicola*. A total of 3473 single-copy orthologous genes were identified, facilitating phylogenetic reconstruction among the species (Figure 2B).

### 3.5. Comparative Analysis and Average Nucleotide Identity (ANI) Value Analysis

Analysis revealed significant genetic collinearity between the *D. carpinicola* genome and two other chromosome-scale genomes (Figure 2C). *D. carpinicola* showed a higher level of collinearity with *Hypoxylon fragiforme*, accounting for 69.53% of the collinear genes (14,503/20,858 genes). The collinearity between *D. carpinicola* and *T. thermophila* was lower, with only 23.28% collinear genes (4149/17,823 genes). The following *D. carpinicola* contigs were suggested to be part of the same chromosome: contig 4 and 8; contig 6, 13, and 44; contig 5, 20, and 45; contig 42 and 46; contig 18 and 34; and contig 7, 10, and 43. A total of 46 *D. carpinicola* contigs showed collinearity with 11 *H. fragiforme* chromosomes

*D. carpinicola* is a newly identified fungal species to avoid confusion with other similar species. The ANI Value Analysis is also gradually applied to the genetic identification of eukaryotic species [33]. Here, the ANI values were used to determine the genetic association among the strains in the *D. carpinicola* genus. The genomes of 12 representative Ascomycetes were extracted and compared with those of *D. carpinicola* (Figure 2D). The *D. eschscholtzii* and *D. bambusicola* ANI values displayed the highest similarity of 84.12%. The ANI value between *D. carpinicola* and *D. eschscholtzii* was 82.05%, indicating high similarity, while that between *D. carpinicola* and *D. bambusicola* was 81.21%. The ANI values of the *H. fuscum*, *Hypoxylon crocopeplum*, *H. fragiforme*, *J. Minutella*, and *Annulohypoxylon nitens* species exceeded 77%. Therefore, the ANI identification results were consistent with the phylogenetic tree.

### 3.6. Gene Cluster Analysis

As shown in Table S4, 50 genes were associated with secondary metabolites, among which 13 are annotated in the MIBIG database, and 3 showed 100% similarity. Of the identified gene regions, 19 were involved in non-ribosomal peptide synthase (NRPS) or NRPS-like pathways, 26 were related to type I polyketide synthase (T1PKS), 4 were associated with indole, 8 impacted terpene biosynthesis, and 1 was related to betalactone and type III polyketide synthase (T3PKS). Three 100% similarity annotated genes were responsible for dihydrolysergic acid (DHLA), pyranonigrin E, and dimethylcoprogen synthesis (Figure 2E). Furthermore, the core biosynthetic genes, additional biosynthetic genes, transport-related genes, regulatory genes, and resistance were marked by distinct colors.

### 3.7. The CAZymes Analyze in the D. carpinicola Genome

**Figure 3:**
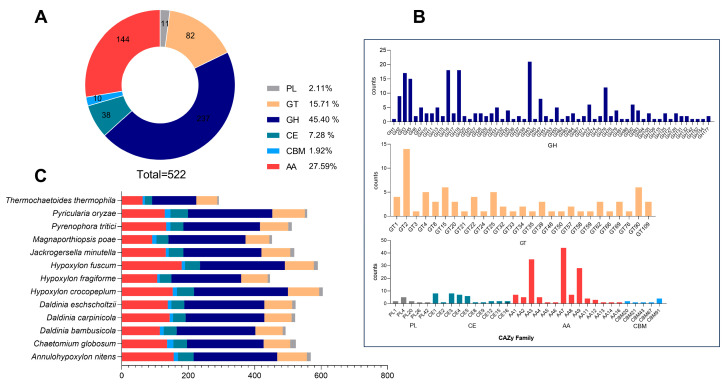
The CAZymes analyze in the *D. carpinicola* Genome. (**A**) The CAZyme category distribution in *D. carpinicola*. (**B**) The CAZyme family genes in *D. carpinicola*. (**C**) The number of distinct orthologous gene types in each fungal species was calculated and represented using different colors.

This study identified 522 potential CAZyme genes in the *D. carpinicola* genome. These included 237 glycoside hydrolases (GHs, 45.40%), 82 glycosyl transferases (GTs, 15.71%), 11 polysaccharide lyases (PLs, 2.11%), 38 carbohydrate esterases (CEs, 7.287%), 144 auxiliary activities enzymes (AAs, 27.59%), and 10 carbohydrate-binding modules (CBMs, 1.92%) (Figure 3A). Figure 3B shows the top 5 GH family genes (21 GH43, 18 GH16, 18 GH18, 17 GH5, and 15 GH3) involved in hemicellulose digestion, contributing to effective degradation in organisms. In addition to GH CAZymes, the *D. carpinicola* was also abundant in ligninolytic enzymes, suggesting a capacity to degrade both plant cell wall polysaccharides and lignin. The genome comprised seven laccase (AA1), five lignin peroxidase (AA2), and thirty-five auxiliary activity (AA3) encoding genes.

As depicted in Figure 3C, the *D. carpinicola* CAZyme profiles were compared with those of 12 other fungi. The result revealed that *D. carpinicola* possessed a higher abundance of CAZyme-encoding genes, with the GH and AA families accounting for a relatively high proportion among those 13 fungi. Moreover, the gene count distribution patterns in the *D. carpinicola* genome exhibited a striking correlation with those observed in the *D. eschscholtzii* genome.

### 3.8. Transcriptome Analysis

**Figure 4:**
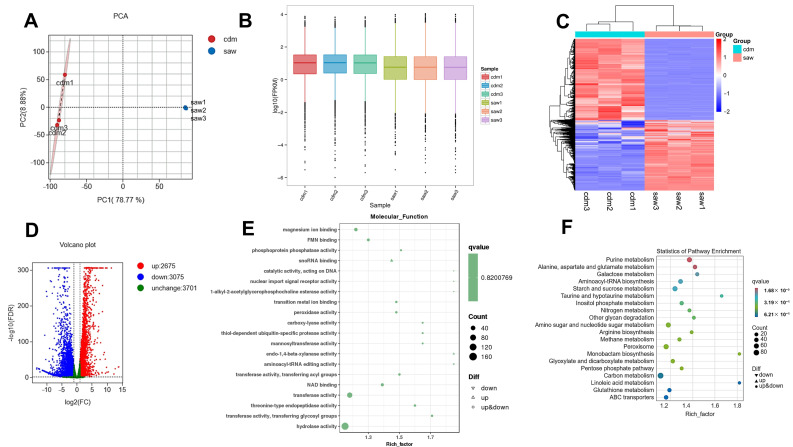
Transcriptome analysis of the different *D. carpinicola* cultures. (**A**) PCA analysis of the two principal components (PC1 and PC2). Different coordinates represent different principal components, percentage represents the contribution value of corresponding principal components to sample differences. (**B**) In the box plot, the horizontal axis denotes the various samples, while the vertical axis signifies the logarithm of the sample expression levels in the FPKM units. (**C**) A heatmap illustrated the overall difference in gene expression in saw culture compared with cdm culture. The X-axis represents each group, and the Y-axis represents the quantitative value of genes standardized by Z-score after hierarchical clustering. (**D**) The volcano plots of the DEGs. Horizontal coordinate represents the logarithmic value of the difference multiple of DEGs. The ordinate represents the negative value of the statistical significance of the change in gene expression. (**E**) The GO enrichment analysis of the DEGs in molecular function. Abscissa represents rich factor, and ordinate represents the GO enrichment pathways. (**F**) The KEGG enrichment analysis of the DEGs. Abscissa represents rich factor, and ordinate represents the KEGG enrichment pathways.

The transcriptomic data have been meticulously curated and subsequently uploaded to the National Center for Biotechnology Information (NCBI) repository under the BioProject Accession Number: PRJNA1209598. In transcriptome analysis, the cdm group acted as the control group and the saw group served as the treatment group. The PCA analysis indicated minor differences between the cdm and saw repeat samples, suggesting a low repeatability error for these samples. Furthermore, it illustrated a clear separation between the cdm and saw groups in the PC1 score plots, indicating a significant difference between the two groups (Figure 4A). The box plot (Figure 4B) illustrated that gene expression levels across the six samples were similarly dispersed, ranging from −6 to 4. It was evident that the parallel samples in the cdm and saw groups exhibited both strong correlations and significant differences.

The transcriptomic heatmap was analyzed to evaluate the DEGs clustering in the samples. The results indicated that the DEGs in the parallel samples of the two groups displayed significant correlation, substantial differences, and clear enrichment (Figure 4C). The transcriptome volcano plot showed that, compared with the cdm culture, the sawdust culture exhibited a total of 5750 DEGs, of which 2675 genes were up-regulated and 3075 were down-regulated (Figure 4D).

The DEGs functions were examined via GO enrichment analysis of the saw and cdm strain groups. Components were compared with cdm cultivation, sawdust cultivation regulated proteasome core complex, beta-subunit complex, peroxisomal matrix, and proteasome core complex, and alpha-subunit complex in cellular (Figure S1a). Additionally, sawdust cultivation regulated small molecule catabolic process, cellular carbohydrate biosynthetic process, carbohydrate biosynthetic and metabolic process, and cell development in cellular component in biological process (Figure S1b). Figure 4E indicated that sawdust cultivation regulated activity of various enzymes, including phosphoprotein phosphatase activity, peroxidase activity, carboxy-lyase activity, transferase activity, and hydrolase activity.

The KEGG enrichment analysis revealed the most significantly enriched pathways when comparing the two groups, including galactose, starch, sucrose, amino sugar, nucleotide, and carbon metabolism, as well as the pentose phosphate pathway and the degradation of other glycans (Figure 4F). Furthermore, the analysis also highlighted certain secondary metabolites such as taurine, hypotaurine, and linoleic acid metabolism. The 130 figures showed the detailed influence of the enzymes and the gene expression regulation information for all 130 metabolic pathways, as shown in the transcriptomic KEGG map file of Supplementary Materials.

### 3.9. Metabolomics Analysis

**Figure 5:**
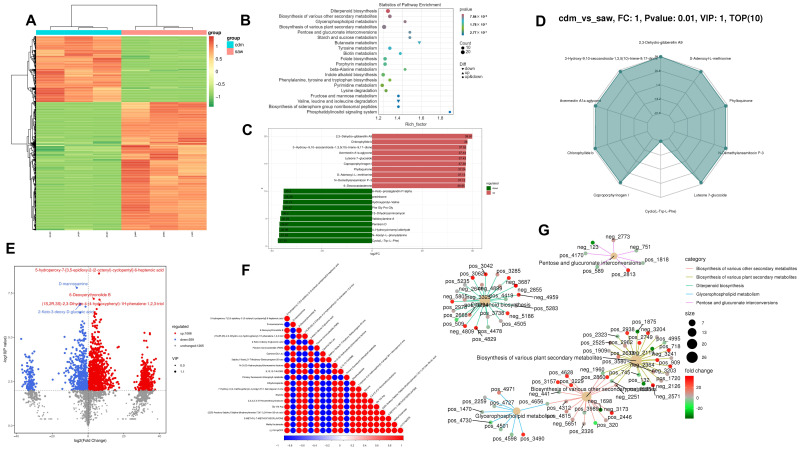
Metabolomics analysis of the different *D. carpinicola* cultures. (**A**) The heatmap of the *D. carpinicola* metabolomic analysis. The X-axis represents each sample, and the Y-axis represents the quantitative value of metabolites standardized by Z-score after hierarchical clustering. (**B**) The KEGG enrichment analysis of the DEMs. Abscissa represents rich factor, and ordinate represents the enrichment pathways. (**C**) Each column is labeled with the corresponding metabolite name, while the column length represents logFC, showing only the 10 substances with the highest differential metabolite levels. (**D**) A radar map of the DEMs. The grid lines represent log2FC, while the green shaded area denotes the log2FC connections for each metabolite. (**E**) The volcano plots of the DEMs. Horizontal coordinate represents the logarithmic value of the difference multiple of DEMs. The ordinate represents the negative value of the statistical significance of the change in metabolites. (**F**) The correlation between the top 20 DEMs and their *p*-values, ordered from smallest to largest, as determined by the *t*-test. (**G**) The light-yellow node represents the pathway, with smaller connected nodes indicating specific metabolites annotated to the pathway.

The metabolomic data have been meticulously compiled and are presented in the Supplementary Materials. In the metabolomics analysis, the cdm group acted as the control group and the saw group served as the treatment group. A total of 41.91 Gb of clean data were obtained from metabolome analysis, and 11,212 peaks were detected, of which 2912 metabolites were annotated. The metabolomic heatmap was analyzed to evaluate the DEGs clustering in the samples. The results showed significant correlations, substantial differences, and clear enrichment between the DEMs of the two parallel sample groups (Figure 5A). OPLS-DA analysis showed that the model was stable and reliable, making it suitable for DEMs screening (Figure S2a).

KEGG enrichment analysis indicated that the different metabolic pathways were primarily associated with secondary metabolite synthesis (diterpenoid, indole alkaloid, and folate biosynthesis and porphyrin and biotin metabolism), carbohydrate metabolism (pentose and glucuronate interconversions, starch, sucrose, fructose, and mannose metabolism), amino acid metabolism (phenylalanine, tyrosine, and tryptophan biosynthesis, tyrosine and beta-alanine metabolism, and lysine, valine, leucine and isoleucine degradation), siderophore-group non-ribosomal peptide biosynthesis, and the phosphatidylinositol signaling system (Figure 5B). The 91 figures provided a detailed overview of all 91 metabolic pathways, as shown in the metabolomic KEGG map file of Supplementary Materials.

As Figure 5B illustrats, compared with the cdm culture, the sawdust culture reduced the D-glucose (extracellular) and acetate and promoted the transition from arbutin and salicin (extracellular) to arbutin 6-phosphate and salicin 6-phosphate during glycolysis/gluconeogenesis. The sawdust culture down-regulated the terpendole G, penitrem D, and lolitriol levels during indole diterpene alkaloid biosynthesis, while up-regulating that of 3-geranyigeranylindole and paspalinine. Furthermore, the sawdust culture down-regulated the pimelate, 7.8-diaminononanoate, dethiobiotin, L-lysine, N6-d-biotinyltne(biocytin), tetranorbiotin, and bisnorbiotin levels during biotin metabolism, while up-regulating that of 8-Amino7-oxononanoate and biotin. Compared with the cdm culture, the sawdust culture down-regulated the 5,6,7,8-te trahydrofolate (THF) and 5-formyl-THF levels during folate biosynthesis while up-regulating that of 5-formimino-THF, 5,10-Methylene-THF, 7,8-Dihydrofolate, and folate were.

A comparison between the top 10 metabolites in each group showed both up-regulation and down-regulation after log conversion (Figure 5C). The up-regulated metabolites included 2,3-Dehydro-gibberellin A9, Chlorophyllide b, 3-Hydroxy-9,10-secoandrosta-1,3,5(10)-triene-9,17-dione, Avermectin A1a aglycone, Luteone 7-glucoside, Coproporphyrinogen I, Phylloquinone, S-Adenosyl-L-methionine, and N-Demethylansamitocin P-3, 6-Deoxocastasterone. The down-regulated metabolites included 6-Keto-prostaglandin F1alpha, prednisone, Hydroxyprolyl-Valine, Phe, Gly, Pro, Gly, 13-Dihydrocarminomycin, Validoxylamine A, Penitrem D, 4-Hydroxycinnamyl aldehyde, N-Acetyl-L-phenylalanine, and Cyclo(L-Trp-L-Phe).

The quantitative DEMs values were used to calculate their ratios, and the top 10 metabolites with the highest absolute log2FC were selected to create the radar chart (Figure 5D). These metabolites included 2,3-Dehydro-gibberellin A9, 3-Hydroxy-9,10-secoandrosta-1,3,5(10)-triene-9,17-dione, S-Adenosyl-L-methionine, Avermectin A1a aglycone, Phylloquinone, Chlorophyllide b, N-Demethylansamitocin P-3, Coproporphyrinogen I, Luteone 7-glucoside, and Cyclo(L-Trp-L-Phe).

The Z-score plot shows the top 30 differentiated metabolites sorted according to the *p*-values (Figure S2b). These metabolites were more extensive and more favorable for screening target metabolites. As the metabolome volcano plot illustrated, compared with the cdm culture, the sawdust culture exhibited a total of 1647 DEMs, of which 1088 were up-regulated and 559 were down-regulated. The most significant DEMs were annotated (Figure 5E), which showed synergistic or mutually exclusive relationships. Pearson’s correlation coefficients between the metabolite pairs were used for DEMs correlation analysis to evaluate the metabolite consistency and trends (Figure 5F). A positive correlation was evident between D-mannosamine, 2-Keto-3-deoxy-D-gluconic acid, Gamma-Glu-Leu, 3alpha, 11beta, 21-Trihydroxy-5beta-pregnan-20-one, and the primary fluorescent chlorophyll catabolite showed a strong correlation, while 5-hydroperoxy-7-[3,5-epidioxy-2-(2-octenyl)-cyclopentyl]-6-heptenoic acid showed a negative association.

Figure 5G displays the enrichment factors for the metabolites associated with diverse secondary plant metabolite biosynthesis, diterpenoid biosynthesis, glycerophospholipid metabolism, and pentose and glucuronate interconversions. Figure S2c illustrates the enrichment factors for other pathways.

### 3.10. Combined Transcriptomic and Metabolomic Analysis

**Figure 6:**
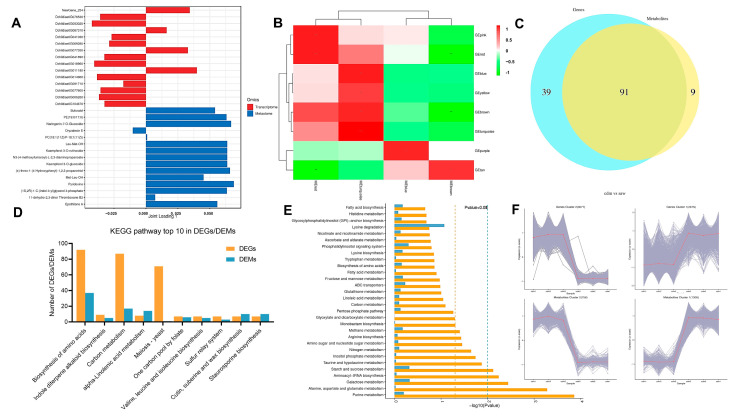
The combined transcriptomic and metabolomic analysis of the different *D. carpinicola* cultures. (**A**) Positive joint loading: This indicates a positive variable correlation between the two omics data. Negative joint loading: This indicates a negative variable correlation between the two omics data. The absolute joint loading value can reflect the importance or influence of the variable in the model and may be a key regulatory factor or biomarker. (**B**) The heatmap color gradient indicates the strength of the correlation between the transcriptomic and metabolomic modules and is used for gene and metabolite cluster analysis. (**C**) The blue section illustrates the metabolic pathways enriched with DEGs at the transcriptional level. In contrast, the yellow section depicts the metabolic pathways enriched with DEMs identified through metabolomic analysis. (**D**) A map showing the top 10 pathways with the most DEGs/DEMs. (**E**) A map showing the top 30 pathways displaying the most significant enrichment. (**F**) The trend chart, where the horizontal axis represents the differential group, while the vertical axis shows the normalized expression of all the DEGs or DEMs in this group. The gray lines represent the expression level of the type of DEGs or metabolite in each sample, while the red lines denote the average expression level.

The top fifteen DEGs and DEMs were screened in the first two load value length dimensions to illustrate the most significant correlations (Figure 6A). The transcriptomic and metabolomic data were analyzed via WGCNA dimensionality reduction. The genes and metabolites were divided into different color modules for clustering and correlation analysis (Figure 6B). A significant positive correlation was observed between the asterisk-labeled dark red and dark green metabolite modules and their corresponding transcriptomic modules.

The Venn diagram revealed that 130 pathways were associated with transcriptomic DEGs, while 100 were related to metabolomic DEMs, showing overlapping between 91 pathways (Figure 6C). The top ten KEGG pathways with the highest number of identified DEGs and DEMs were visualized, including amino acid, indole diterpene alkaloid, valine, leucine, isoleucine, cutin, suberine, and wax biosynthesis, carbon and alpha-linolenic acid metabolism, yeast meiosis, and one-carbon pool via folate (Figure 6D). The top 30 most significantly enriched pathways complemented and supported analysis result of the top 10 KEGG pathways (Figure 6E). The intersection of the metabolic pathways identified by the two statistical KEGG methods included amino acid biosynthesis and carbon metabolism.

The metabolites and genes displaying the same change trend were used for correlation analysis. Compared to the cdm group, 3077 gene clusters corresponding to 756 metabolites exhibited decreased expression levels, while 2675 gene clusters corresponding to 1335 metabolites showed increased expression levels in saw group (Figure 6F).

**Figure 7:**
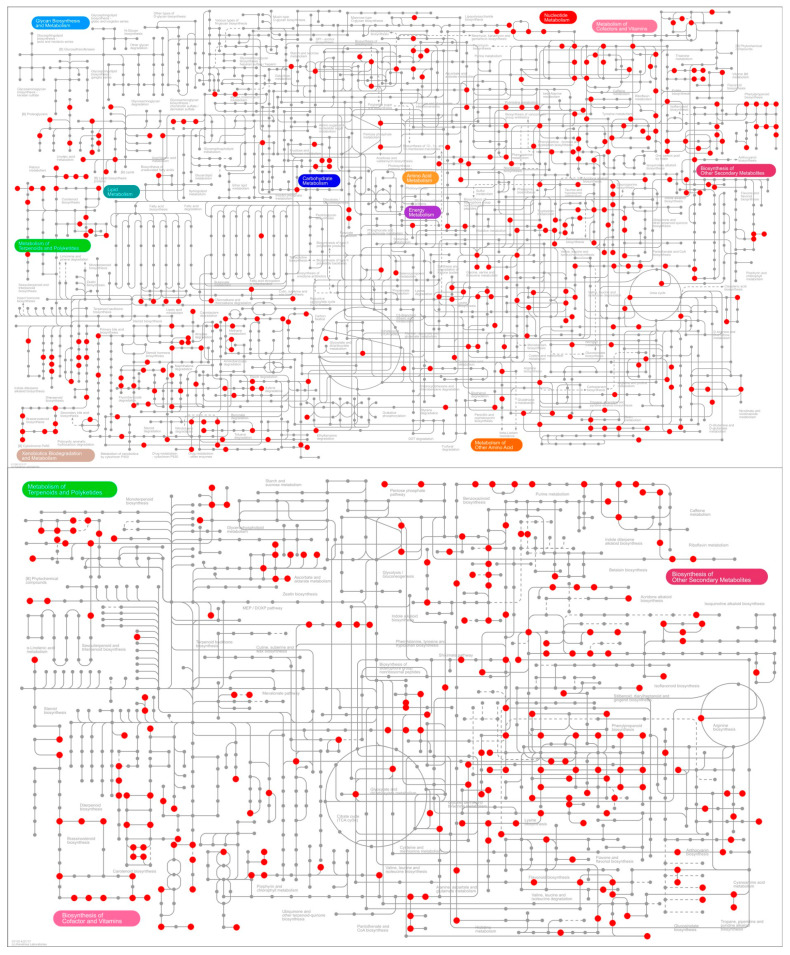
Ipath map of combined transcriptomic and metabolomic analysis. The nodes denote various compounds, while boundaries signify distinct enzymatic reactions. The red dots indicate reactions where DEGs/DEMs were identified, while the variety of lines illustrates different metabolic pathways or functions.

Figure 7 provided a visual representation of the metabolic pathways, outlining a global overview of the biosynthesis of secondary metabolites and key regulatory pathways. The Ipath map showed the detailed sites of the saw influence on the metabolic and secondary metabolic pathways of *D. carpinicola* relative to the cdm groups.

## 4. Discussion

Third-generation and high-throughput sequencing technologies were utilized to assemble the *D. carpinicola* reference genome and elucidate its molecular mechanisms. The gene assembly was optimal, with an N50 of 1.323 Mb, which was consistent with the genome sequencing results of many fungi, such as *Leucocalocybe mongolica* [34], *Cordyceps guangdongensis* [35], and *Auricularia heimuer* [36]. These genomes, characterized by N50 values surpassing 1 Mb, have been reported in published research. Further analysis revealed that 463 genes belonged to the P450 family, while 50 were associated with the secondary metabolism gene cluster including NRPS or NRPS-like, T1PKS, indole, terpene, betalactone, and T3PKS. Secondary metabolites, such as indole alkaloids, porphyrins, biotin, and folic acid, possess high medicinal value, indicating their significant potential for various applications [10].

A previous study found that a total of 56 gene clusters related to secondary metabolites were predicted in *Oudemansiella raphanipes* (Changgengu), including terpenes, NRPS or NRPS-like, T1PKS, indole, siderophore, and fungal-RiPP-like [37]. These beneficial secondary metabolites are in line with people’s production needs. In the present study, DHLA, pyranonigrin E, and dimethylcoprogen synthesis were annotated with 100% similarity in the MIBIG database. DHLA is a crucial bioactive substance that serves as a pivotal intermediary in the ergot alkaloid biosynthetic pathway [37,38]. However, using DHLA as a possible alternative pathway for synthesizing ergot alkaloids requires further investigation [37]. The cloA gene, originating from a sorghum ergot fungus (*Claviceps africana*) that synthesizes a DHLA derivative, was cloned and expressed as a festuclavine-accumulating *Neosartorya fumigate* mutant [39]. Pyranonigrin E is a polyketide synthase-non-ribosomal peptide synthetase (PKS-NRPS) hybrid metabolite produced by *Aspergillus niger*. Genome mining methods were used to activate the PKS-NRPS gene cluster in *Aspergillus niger* ATCC 1015. The expression of its specific PynR transcriptional activator yielded a new pyranonigrin compound, namely pyranonigrin E [40]. Previous studies involving pyranonigrin E isolation and biosynthesis have highlighted its excellent antioxidant properties and unique chemical structure. Dimethylcoprogen, a novel lateral ferritin originally isolated from *Alternaria longipes* (ATCC 26293) [41], is an N-α-dimethylated analog of coprogen, neocoprogen I, and isoneocoprogen I. Dimethylcoprogen biosynthesis is typically examined by analyzing gene clusters. Specifically, NRPS genes associated with dimethylcoprogen biosynthesis have been identified in the *Ceratocystis destructans* fungal genome [42]. Furthermore, several genes have been annotated in the genomes of edible fungi, such as pyranonigrin E derived from *Aspergillus niger* [43] and dimethylcoprogen from *Alternaria alternata* [39].

CAZymes are vital for the survival of wood-rotting mushrooms, enabling them to flourish in carbohydrate-rich environments, particularly those high in lignocellulose and cellulose [33]. *D. carpinicola*, a type of wood-rotting fungus, contained 522 candidate CAZyme genes, with GHs the most abundant, which was three-fold higher than the GT levels. The ability of *D. carpinicola* to break down lignocellulose, along with the predominant role of its GH-related genes in starch degradation, likely underpinned its survival in carbohydrate-rich environments [44]. The GH43 family predominantly catalyzes xylan and arabinoxylan hydrolysis [45]. The GH16 family specializes in cleaving diverse β-linked polysaccharides, except for cellulose [46]. The GH5 family encompasses 21 distinct activities, mainly featuring endo/exo-β-1,4-glucanase, β-mannanase, and mannosidase, which influences β-linked oligosaccharides and polysaccharides [47]. The GH3 family comprises 11 distinct enzymatic activities, mainly featuring β-glucosidase and β-xylosidase, which catalyzes oligosaccharide conversion into monosaccharides [48]. The GH18 family specializes in cleaving β-linked N-acetylglucosamine moieties in chitin, chitooligosaccharides, peptidoglycan, and N-glycans. Additionally, the genome contained two GH6 and five GH7 genes essential for enzymatic cellulose chain cleavage. They function as either endoglucanases or cellobiohydrolases, mainly generating cellobiose from the ends of cellulose chains, regardless of whether it is the reducing or non-reducing end [49].

The AAs are second in number and play an important role in the breakdown of carbohydrates. Multicopper oxidases, known as AA1 enzymes, are crucial for biochemical lignin breakdown. Class II lignin-modifying peroxidases (AA2) are essential for lignin degradation, serving as distinctive biomarkers that differentiate between white-rotting and brown-rotting fungi [33]. AA3 enzymes assist other AA family enzymes or GHs in lignocellulose degradation. AA9 enzymes, characterized as copper-dependent lytic polysaccharide monooxygenases, facilitate oxidative cellulose cleavage, converting polysaccharides and oligosaccharides into monomeric sugars [50]. The *D. carpinicola* genome contained 28 AA9 genes, indicating its substantial degradative potential towards lignocellulosic biomass. Additionally, *D. eschscholtzii* displayed a close evolutionary relationship with *D. carpinicola*, also containing CAZymes and genes that encode secreted peptidases and degrade the structural proteins in plant cell walls [51]. Meanwhile, *D. carpinicola* exhibited a similar number of CAZyme genes to *D. eschscholtzii*.

The prevailing theory posits that the transformation of organic matter into high molecular weight humic substances typically proceeds via two distinct pathways. Polymeric organic materials, such as lignin, cellulose, and hemicellulose, are progressively broken down into humic substance precursors via a sequential process [52]. Fungal communities accelerate this process by efficiently producing a diversity of extracellular enzymes [53,54]. Low molecular weight compounds are extracted from organic matter in the alternative pathway, which is readily accessible to a broad spectrum of microorganisms [55]. The impact of diverse carbon sources on the physiological metabolism of *D. carpinicola* was investigated. *D. carpinicola* was originally isolated from the environment of chestnut wood, a habitat characterized by specific ecological and nutritional conditions that may have shaped the fungus’s metabolic and growth characteristics. In parallel, the utilization of wood chip-based culture substrates has become a prevalent practice within most modern edible mushroom industries. This substrate’s popularity can be attributed to its ability to mimic the natural habitat of many edible fungi, thereby providing an optimal growth medium that aligns with the industry’s pursuit of efficiency and yield [56]. Prior research has elucidated that fungi perceive alterations in the extracellular milieu via intricate signal transduction pathways. These pathways are responsive to a variety of environmental cues, including nutrient paucity, the transformation and utilization of matter, and fluctuations in population density. Such perceptions trigger the activation of biosynthetic gene clusters responsible for producing secondary metabolites, thereby yielding a plethora of bioactive compounds [56]. Conversely, in artificial media characterized by an abundance of nutrients and a stable environment, the intricate regulatory mechanisms that govern secondary metabolite synthesis may remain dormant, leading to a diminution in the production of these metabolites. Compared with sawdust, sucrose as a culture carbon source accelerated *D. carpinicola* cultivation, suggesting that *D. carpinicola* was more efficient in utilizing small-molecule carbon sources for growth. The main metabolic pathways were concentrated in glycolysis/gluconeogenesis and the citrate cycle. Research has also shown that fungi and *Saprolegniales* strains prefer certain low molecular carbon sources despite the abundant availability of high molecular-weight carbon sources in ecosystems [52]. Compared to sucrose, sawdust as a carbon source more effectively activated the metabolic pathways of certain secondary metabolites, including diterpenoids, biotin, indole alkaloids, and folic acid. This may also be attributed to the fact that sawdust, as complex natural organic materials, contain not only cellulose and hemicellulose but also a large amount of lignin and trace substances like resins and pigments [57]. Similarly, previous studies have shown that fungal communities adapt to various labile carbon sources, including glycine and sucrose, potentially specializing in metabolic compound degradation and producing more secondary metabolites [58].

The utilization of diterpenoids, biotin, indole alkaloids, and folic acid has been the subject of numerous applications, and concurrent research endeavors have been directed towards devising methodologies to augment their biosynthetic yields. Diterpenoids have a variety of pharmacological effects, including anti-inflammatory, anti-ulcer, hypoglycemic, hypolipidemic, and anticoagulant, as well as bacteriostatic effects. Engineering the microenvironment of Cytochrome P450 enzymes enhanced the production of diterpenoids in *Saccharomyces cerevisiae* [59]. Biotin serves not only as a coenzyme in carboxylation reactions but also finds extensive application in industries such as feed, food, and pharmaceuticals. Treating *Bacillus phaericus* with chemical mutagens N-methyl-N’-nitro-N-nitrosoguanideine improved the biotin production [60]. Indole alkaloids, one of the largest classes of alkaloids, prove to be valuable structural moieties in pharmaceuticals. A modular assembly approach to tetrahydrocarboline-type indole alkaloids significantly enhances the production efficiency of indole alkaloids [61]. Folic acid, an essential micronutrient, serves as a medicinal treatment for various conditions, including cancer, anemia, cardiovascular diseases, and neural tube defects. Furthermore, the model organism *Escherichia coli* has been genetically modified to overproduce folate through the deletion of the pyruvate kinase gene [62]. Therefore, research on different carbon sources for culturing the *Daldinia* genus represents a novel approach aimed at enhancing the production of beneficial secondary metabolites.

## 5. Conclusions

This study provides the *D. carpinicola* reference genome for further functional studies. Transcriptomic and metabolomic analysis of *D. carpinicola* cultured on different carbon sources elucidates the mechanisms underlying its carbon source utilization. This study is the first to map the entire genome of the newly identified *D. carpinicola* wood-rotting fungi. *D. carpinicola* is cultured and sequenced using a Nanopore third-generation sequencer, yielding 46 contigs with N50 lengths of 1.323 Mb. A proprietary database is used for functional *D. carpinicola* genome annotation. Comparative genomic, evolutionary, and CAZyme analysis provides substantial insight into the *D. carpinicola* taxonomic status, survival mechanisms, and functional abilities. The genome contains 463 genes belonging to the P450 family. Furthermore, 50 secondary metabolite synthesis genes are identified, 13 of which are known secondary metabolite synthesis genes, providing valuable information for the development of metabolite applications. Compared with sawdust, *D. carpinicola* more effectively utilizes sucrose as a carbon source to enhance its growth by activating key metabolic pathways such as glycolysis/gluconeogenesis, the citrate cycle, and the pentose phosphate pathway. However, compared to sucrose, using sawdust as a carbon source activates the biosynthesis of amino acids and various secondary metabolites in *D. carpinicola*, including diterpenoid, indole alkaloid, and folate biosynthesis and porphyrin and biotin metabolism. The genomic, transcriptome, and metabolome analysis in this study establishes a theoretical basis for research and applications in biological processes, demonstrating a strategy to modulate the production of secondary metabolites by altering its carbon sources in *D. carpinicola*.

## Figures and Tables

**Table 1:** Genome assembly features of the *D. carpinicola*.

Contig Length (bp)	Contigs	Scaffold N50 (bp)	Scaffold N90 (bp)	GC Content (%)	Gaps Number	Library (bp)	Mapped (%)	Properly Mapped (%)	Coverage (%)	Depth (X)
35,934,728	46	1,323,482	550,949	44.81	0	350	81.71	78.97	99.97	74.29

**Table 2:** Characteristics of the gene prediction of *D. carpinicola*.

Content	Number/Length
Gene number	10,596
Gene length (bp)	23,949,977
Average gene length (bp)	2260.28
Exon length (bp)	21,747,637
Average exon length (bp)	670.77
Exon number	32,422
Average exon number	3.06
CDS length (bp)	16,005,309
Average CDS length (bp)	509.17
CDS number	31,434
Average CDS number	2.97
Intron length (bp)	2,202,340
Average Intron length (bp)	100.9
Intron number	21,826
Average Intron number per gene	2.06

## Data Availability

The original contributions presented in this study are included in the article/Supplementary Material. Further inquiries can be directed to the corresponding author(s).

## Supplementary Materials

The following supporting information can be downloaded at: https://www.mdpi.com/article/10.3390/jof11020115/s1, Figure S1: The GO enrichment analysis of the DEGs in cellular component and biological process; Figure S2: Scores (OPLS-DA), Z-score plot and enrichment factors analysis of the different *D. carpinicola* cultures; Table S1: BUSCO assessment statistics of the *D. carpinicola* genome; Table S2: Statistics of comparison results of the *D. carpinicola* genome; Table S3: The NCBI Accession No of the strains; Table S4: Gene cluster prediction results of the *D. carpinicola* genome; Metabolome data; Metabolome KEGG map; Transcriptome KEGG map.
