# Supplementary material for: Multiomic Analysis Provided Insights into the Responses of Carbon Sources by Wood-Rotting Fungi Daldinia carpinicola
Source: J Fungi (Basel). 2025 Feb 4;11(2):115. doi: 10.3390/jof11020115 (PMC11856974; doi:10.3390/jof11020115)
Supplement: Supplementary file 1 [file jof-11-00115-s001.zip › jof-3369885-supplementary/Supplementary figures and tables .pdf]

Supplementary Materials

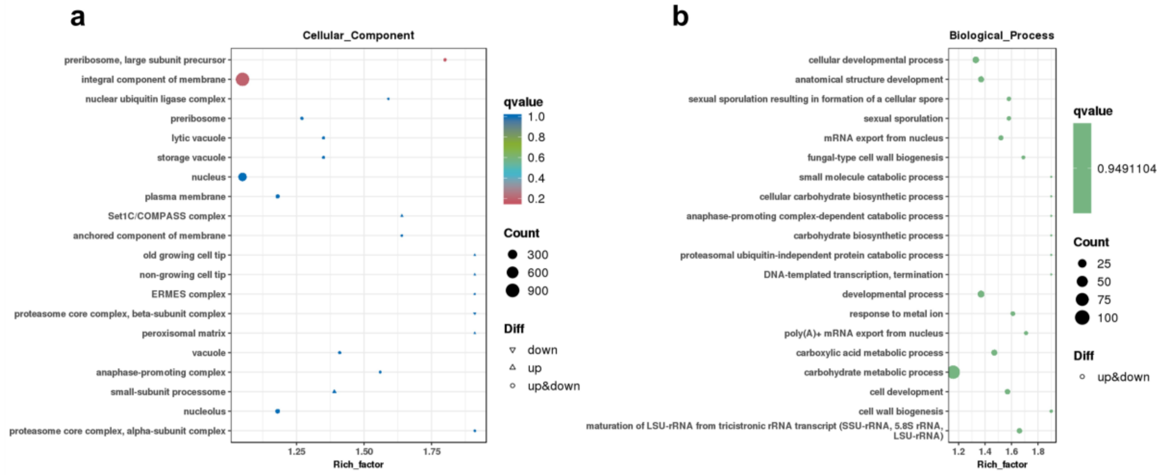

Figure S1. The GO enrichment analysis of the DEGs in cellular component and biological process. (a) The GO enrichment analysis of the DEGs in cellular component. Abscissa represents rich factor, and ordinate represents the GO enrichment pathways. (b) The GO enrichment analysis of the DEGs in biological process. in molecular function Abscissa represents rich factor, and ordinate represents the GO enrichment pathways.

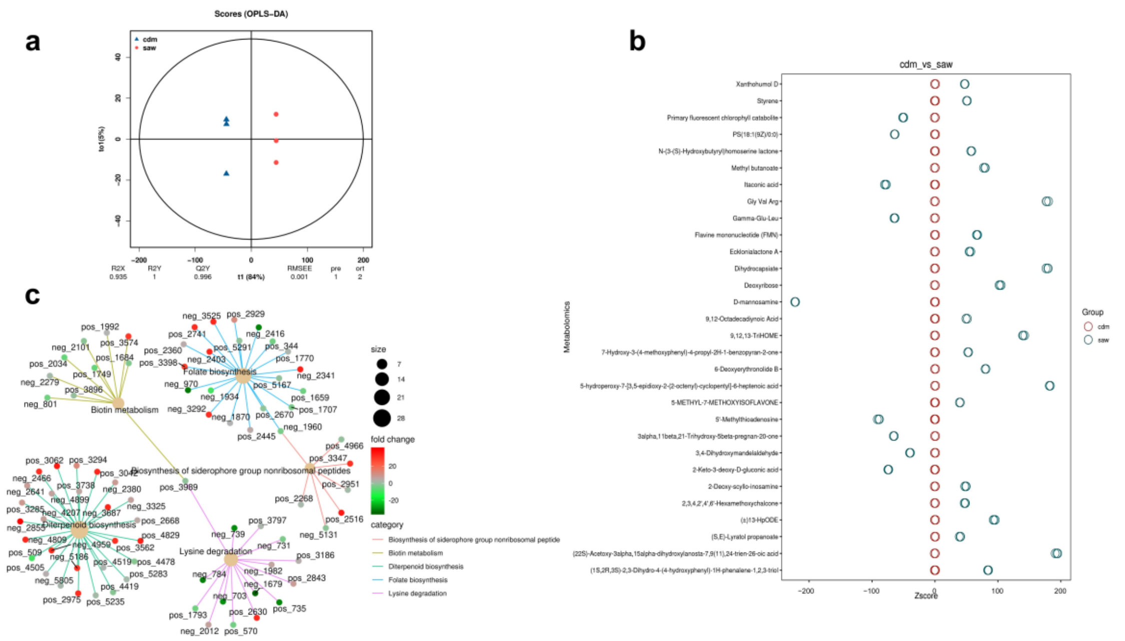

Figure S2. Scores (OPLS-DA), Z-−score plot and enrichment factors analysis of the different *D. carpinicola* cultures. **(a)** The X-axis (t1) represents the prediction component (inter-group difference component) and the Y-axis (to1) represents the orthogonal component (intra-group difference component). R2X and R2Y: the interpretation rate of the model to the x and y matrix. Q2Y: the prediction ability of the model. RMSEE: root mean square error. Pre and

ort: number of predictive and orthogonal components modeled by OPLS-DA. **(b)** The Z-score (standard score) is a value based on the conversion of quantitative values of metabolites to measure the difference deviation of the experimental group from the control group. **(c)** The pathway is represented by a light-yellow node, with smaller connected nodes indicating specific metabolites annotated to that pathway.

Table S1. BUSCO assessment statistics of the *D. carpinicola* genome.

Complete BUSCOs(C)	Complete and single-copy BUSCOs(S)	Complete and duplicated BUSCOs(D)	Fragmented BUSCOs(F)	Missing BUSCOs(M)	Total Lineage BUSCOs
281 (96.90%)	279 (96.21%)	2 (0.69%)	4 (1.38%)	5 (1.72%)	290

Note: Complete BUSCOs: find the complete number of genes; Complete and single-copy BUSCOs: the number of single-copy genes; Complete and duplicated BUSCOs: the number of multiple copy genes; Fragmented BUSCOs: predicted the number of incomplete genes; Missing BUSCOs: no predicted number of genes; Total Lineage BUSCOs: number of fungal conserved gene sets.

Table S2. Statistics of comparison results of the *D. carpinicola* genome.

**Library**	**Mapped(%)**	**Properly mapped(%)**	**Coverage(%)**	**Depth(X)**
350bp	81.71	78.97	99.97	74.29

Note: Library: next generation sequencing library size; mapped(%) : the percentage of Clean Reads mapped to the reference genome among all Clean Reads; Properly mapped(%) : both ends of the sequencing sequence were located on the reference genome and the distance was consistent with the length distribution of the sequencing fragment; Coverage(%): genome coverage; Depth(X) : depth of genome coverage.

Table S3. The NCBI Accession No of the strains.

**Strain name**	**Accession No**
Daldinia carpinicola	JBKKKA000000000
Annulohypoxylon nitens	JAJKHP01
Chaetomium globosum	AAFU01
Daldinia bambusicola	JAJMPD01
Daldinia eschscholtzii	JBANMG01
Hypoxylon crocopeplum	JAJLYL01
Hypoxylon fragiforme	JAJKLD01
Hypoxylon fuscum	JAJLYN01
Jackrogersella minutella	JAJKKJ01
Magnaporthiopsis poae	ADBL01
Pyrenophora tritici-repentis	AAXI01
Pyricularia oryzae	AACU03
Thermochaetoides thermophila	ADUW01

Table S4. Gene cluster prediction results of the *D. carpinicola* genome.

Region	Type	From	To	Most similar known cluster		Similarity
Region 1.1	NRPS-like	674,237	717,220			
Region 1.2	indole	1,175,495	1,192,801			
Region 1.3	NRPS,indole	1,272,042	1,346,032	dihydrolysergic acid	Alkaloid	100%
Region 1.4	T1PKS	1,415,166	1,462,337			
Region 1.5	NRPS-like	1,870,731	1,914,022			
Region 2.1	T1PKS	659,274	703,007			
Region 2.2	NRPS-like	776,915	820,545			
Region 2.3	T1PKS	848,167	896,778	pyranonigrin E	Polyketide	100%
Region 3.1	T1PKS	267,891	316,210			
Region 4.1	NRPS	239,732	284,863			
Region 4.2	indole	381,529	402,071			
Region 7.1	terpene	47,329	68,949	squalestatin S1	Terpene	40%
Region 7.2	T1PKS	280,999	331,175			
Region 9.1	T1PKS	775,545	823,185	solanapyrone D	Polyketide	66%
Region 10.1	T1PKS	70,921	107,155			
Region 10.2	terpene	159,791	181,705			
Region 10.3	NRPS-like,T1PKS	430,501	478,003	swainsonine	Polyketide	85%
Region 11.1	NRPS-like	448,485	491,894			
Region 12.1	NRPS-like	645,663	684,736			
Region 12.2	NRPS-like	710,184	746,625			
Region 12.3	indole	1,673,968	1,695,349			
Region 12.4	T3PKS	1,704,004	1,745,425			
Region 12.5	NRPS	2,209,154	2,264,181			
Region 13.1	T1PKS	530,683	593,208	chaetoviridin E / 11-epichaetomugilin A	Polyketide	16%
Region 14.1	NRPS,T1PKS	143,075	196,803			
Region 14.2	T1PKS	350,424	398,622			
Region 15.1	T1PKS	210,233	257,469			
Region 17.1	terpene	372,205	393,684			
Region 18.1	terpene	699,777	720,871			
Region 19.1	T1PKS	61,704	104,253	wortmanamide A / wortmanamide B	NRP + Polyketide	83%
Region 19.2	terpene	197,343	218,744			
Region 21.1	T1PKS	378,582	426,513	eupenifeldin	Terpene	27%
Region 23.1	T1PKS	34,465	77,558			
Region 23.2	T1PKS	1,850,119	1,897,515			
Region 26.1	NRPS-like	19,796	56,159			
Region 26.2	NRPS	90,094	135,421	dimethylcoprogen	NRP	100%
Region 27.1	T1PKS	13,327	61,341			
Region 34.1	NRPS-like	165,841	209,119			
Region 34.2	T1PKS,terpene	823,108	865,899			
Region 34.3	T1PKS	1,244,847	1,282,140			
Region 34.4	T1PKS	1,446,199	1,537,526	naphthalene	Polyketide	44%
Region 42.1	T1PKS	139,653	173,658			
Region 42.2	T1PKS,terpene	766,675	826,327	neurosporin A	Polyketide	20%

Region 43.1	T1PKS	200,844	249,101			
Region 43.2	betalactone,NRPS	659,150	713,694			
Region 43.3	terpene	1,816,927	1,831,403			
Region 44.1	NRPS-like	264,521	304,179			
Region 44.2	T1PKS,NRPS	1,302,041	1,354,748	wortmanamide A / wortmanamide B	NRP + Polyketide	66%
Region 45.1	T1PKS,NRPS	1,852,618	1,927,910			
Region 46.1	T1PKS,NRPS	395,342	448,215	cytochalasin E / cytochalasin K	NRP + Polyketide:Iterative type I	61%
