# Supplementary material for: Multiomic Analysis Provided Insights into the Responses of Carbon Sources by Wood-Rotting Fungi Daldinia carpinicola
Source: J Fungi (Basel). 2025 Feb 4;11(2):115. doi: 10.3390/jof11020115 (PMC11856974; doi:10.3390/jof11020115)
Supplement: Supplementary file 1 [file jof-11-00115-s001.zip › jof-3369885-supplementary/Metabolome data/BMK220923-BB524-ZX01-0301.data.tar.gz!/input/BMK220923-BB524-ZX01-0301-report.docx]

**北京百迈客样品检测报告**

**项目信息**

| **项目名称** | **真菌基因组+9个转录组代谢组测序分析** | **项目编号** | **BMK220923-BB524-ZX01-03** |
| --- | --- | --- | --- |
| **客户单位** | **青岛农业大学** | | |
| **客户姓名** | **杨鹏** | **合同样品数** | **9** |
| **实验人员** | **张露** | **完成时间** | **2023.04.18** |
| **客户经理** | **乔梦** | | |
| **审核人** |  | **审核时间** |  |

**UHPLC-QTOF-MS非靶标代谢组学检测报告**

# 一、样本基本信息

共收到客户提供的9例样本，实际检测9例样本。

表1. 样本信息表

| **组别** | **A** | **B** | **C** |
| --- | --- | --- | --- |
| 样本数量 | 3 | 3 | 3 |
| 实验检测 | 3 | 3 | 3 |

***注：****①样本收到后，立即保存在-80℃低温冰箱中，直至实验检测；②为了更好地进行实验，对样本编号进行了重新编号，详细见“样本编号对应表”（如果没有重新编号则无该表）。*

# 二、实验信息

## 2.1 仪器及试剂

### 2.1.1 实验试剂

表2. 实验试剂列

| **名称** | **CAS** | **纯度** | **品牌** |
| --- | --- | --- | --- |
| 甲醇（Methanol） | 67-56-1 | LC-MS级 | Merck |
| 乙腈（Acetonitrile） | 75-05-8 | LC-MS级 | Merck |
| L-2-氯苯丙氨酸（2-Chloro-L-phenylalanine） | 103616-89-3 | ≥98% | 上海阿拉丁 |
| 甲酸(Formic acid) | 64-18-6 | LC-MS级 | TCI |

### 2.1.2所用主要仪器平台

1.超高效液相色谱：Waters UPLC Acquity I-Class PLUS

2.高分辨质谱：Waters UPLC Xevo G2-XS QTof

3.色谱柱： Acquity UPLC HSS T3 1.8um 2.1*100mm,Waters;

## 2.2 实验方法

### 2.2.1代谢物提取

1.称取50 mg样本，加入1000μL含有内标的提取液（甲醇乙腈水体积比=2:2:1，内标浓度20mg/L），涡旋混匀30秒；

2.加入钢珠，45Hz研磨仪处理10min，超声10min（冰水浴）；

3.零下20℃ 静置一小时；

4.将样本4℃，12000rpm离心15min；

5.小心地取出500μL上清于EP管中；

6.在真空浓缩器中干燥提取物；

7.向干燥后的代谢物加入160μL 提取液（乙腈水体积比：1:1）复溶；

8.涡旋30秒，冰水浴超声10分钟；

9.将样本4℃，12000rpm离心15min；

10.小心地取出120μL上清于进样瓶，每个样本各取10μL混合成QC样本上机检测。

### 2.2.2上机检测

用于代谢组学分析的液质联用系统由沃特世Acquity I-Class PLUS超高效液相串联沃特世Xevo G2-XS QTof高分辨质谱仪组成所使用色谱柱为购自沃特世的Acquity UPLC HSS T3 色谱柱（1.8um 2.1*100mm）

正离子模式：流动相A：0.1%甲酸水溶液；流动相B：0.1%甲酸乙腈

负离子模式：流动相A：0.1%甲酸水溶液；流动相B：0.1%甲酸乙腈

进样体积：2ul

表3. 液相色谱梯度参数

| **时间(min)** | **流速(μL/min)** | **A%** | **B%** |
| --- | --- | --- | --- |
| **0.0** | 400 | 95 | 5 |
| **0.5** | 400 | 95 | 5 |
| **5.5** | 400 | 50 | 50 |
| **9.0** | 400 | 5 | 95 |
| **10.5** | 400 | 5 | 95 |
| **12.0** | 400 | 95 | 5 |

质谱条件：

沃特世Xevo G2-XS QTof高分辨质谱仪能够在采集软件（MassLynx V4.2,Waters）控制下的MSe模式进行一级、二级质谱数据采集。在每个数据采集循环中，能够同时对低碰撞能量及高碰撞能量进行双通道数据采集。低碰撞能量2V，高碰撞能量区间为10~40V，扫描频率为0.2秒一张质谱图。ESI离子源参数如下：毛细管电压：2500V（正离子模式）或-2000V（负离子模式）；锥孔电压：30V；离子源温度：100℃；脱溶剂气温度500℃；反吹气流速：50L/h；脱溶剂气流速：800L/h；质核比（m/z）采集范围50-1200。

### 2.2.3数据处理

使用MassLynx V4.2采集的原始数据通过Progenesis QI软件做峰提取、峰对齐等数据处理操作，基于Progenesis QI软件在线METLIN数据库及百迈客自建库进行鉴定，同时进行理论碎片识别，母离子质量数偏差100ppm、碎片离子质量数偏差50ppm以内。

## 2.3.实验结果及质控

**1、过程质控**

样品的检测要持续很长时间，尤其是当样本量很大的时候。在检测过程中实时地监控仪器稳定性、信号是否正常就十分重要。及时发现异常，尽早将问题排除，以保证最终采集数据的质量。

1. 仪器稳定性

由图1、图2可以看到，QC样本BPI出峰保留时间和峰面积都重叠很好，说明仪器稳定性很好。

图1.所有QC样本正离子BPI/TIC图

图2.所有QC样本负离子BPI/TIC图

1. 内标响应情况

由图3、4可以看到内标L-2-氯苯丙氨酸在样品中的保留时间和峰面积稳定性很好。说明仪器数据采集稳定性很好。

图3.所有QC样本正离子内标响应

图4.所有QC样本负离子内标响应

**2、数据质控**

色谱质谱联用仪是一个十分复杂精密的系统，在样品检测过程中会受到温度、湿度、震动、电路板老化等客观因素的影响，导致一定程度上响应信号波动。实验人员的操作也会带来不确定的误差。这就需要一系列的质控手段来进行数据处理，以获得高质量的数据。

1. 样本在PCA分析中的呈现

理论上讲，QC样本都是相同的，但是在物质提取、检测分析过程中会有误差，导致QC样品间会有差异。这个差异越小说明整个方法稳定性越好数据质量越高。图5，图6 PCA分布图，可以看出所有样本QC相对集中，组内差异不明显，组间差异明显。

图5.样本正离子PCA得分图

图6.样本负离子PCA得分图

## 2.4 实验小结

1、 样本质量：良好；
2、 实验方法：良好；
3、 系统稳定性：良好

## 2.5 参考文献

1.	Dunn WB, Broadhurst D, Begley P, et al. Procedures for large-scale metabolic profiling of serum and plasma using gas chromatography and liquid chromatography coupled to mass spectrometry. *Nat Protoc* 2011; **6**(7): 1060-83. （血清、血浆样本前处理方法参考文献）

2.	Want EJ, Wilson ID, Gika H, et al. Global metabolic profiling procedures for urine using UPLC-MS. *Nat Protoc* 2010; **5**(6): 1005-18. （尿液样本前处理方法参考文献）

3. Wang J, Zhang T, Shen X, et al. Serum metabolomics for early diagnosis of esophageal squamous cell carcinoma by UHPLC-QTOF/MS[J]. *Metabolomics*, 2016, **12**(7):116.（样品检测方法参考文献）

4.	William O. Slade,Emily G. Werth,Evan W. McConnell,Sophie Alvarez,Leslie M. Hicks. Quantifying Reversible Oxidation of Protein Thiols in Photosynthetic Organisms[J]. Journal of The American Society for Mass Spectrometry,2015,26(4).（数据处理方法参考文献）

5. Tetrahydrobiopterin and alkylglycerol monooxygenase substantially alter the murine macrophage lipidome[J]. Proceedings of the National Academy of Sciences of the United States of America,2015,112(8).（数据处理方法参考文献）

6.	Kuhl C, Tautenhahn R, Böttcher C, et al. CAMERA: An Integrated Strategy for Compound Spectra Extraction and Annotation of Liquid Chromatography/Mass Spectrometry Data Sets[J]. *Analytical Chemistry*, 2012, **84**(1):283-9.（数据处理方法参考文献）
