# Supplementary material for: Multiomic Analysis Provided Insights into the Responses of Carbon Sources by Wood-Rotting Fungi Daldinia carpinicola
Source: J Fungi (Basel). 2025 Feb 4;11(2):115. doi: 10.3390/jof11020115 (PMC11856974; doi:10.3390/jof11020115)
Supplement: Supplementary file 1 [file jof-11-00115-s001.zip › jof-3369885-supplementary/Metabolome data/BMK220923-BB524-ZX01-0301.data.tar.gz!/input/neg-total.pdf]

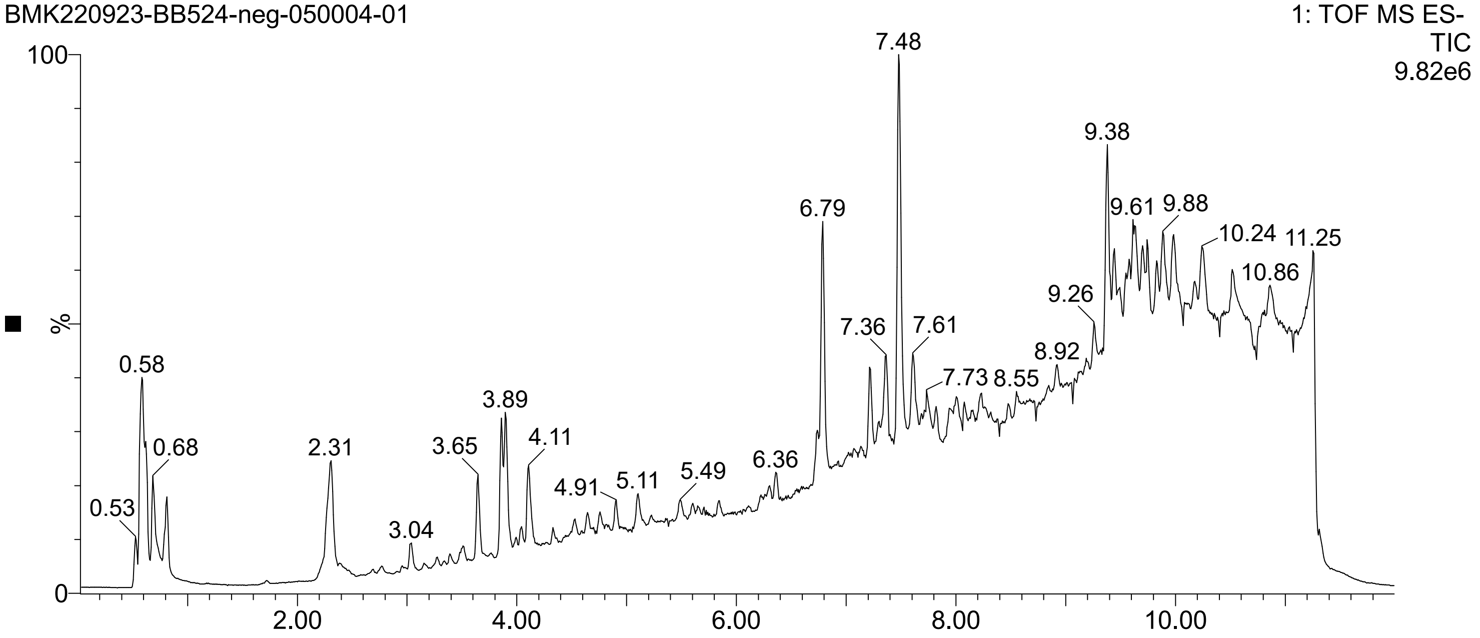
BMK220923-BB524-neg-050004-01
1: TOF MS ES-
TIC
9.82e6
100
%
0
7.48
9.38
6.79
9.61
9.88
10.24
11.25
10.86
9.26
7.36
7.61
8.92
7.73
8.55
0.58
3.89
0.68
2.31
3.65
4.11
6.36
5.49
4.91
5.11
0.53
3.04
2.00
4.00
6.00
8.00
10.00

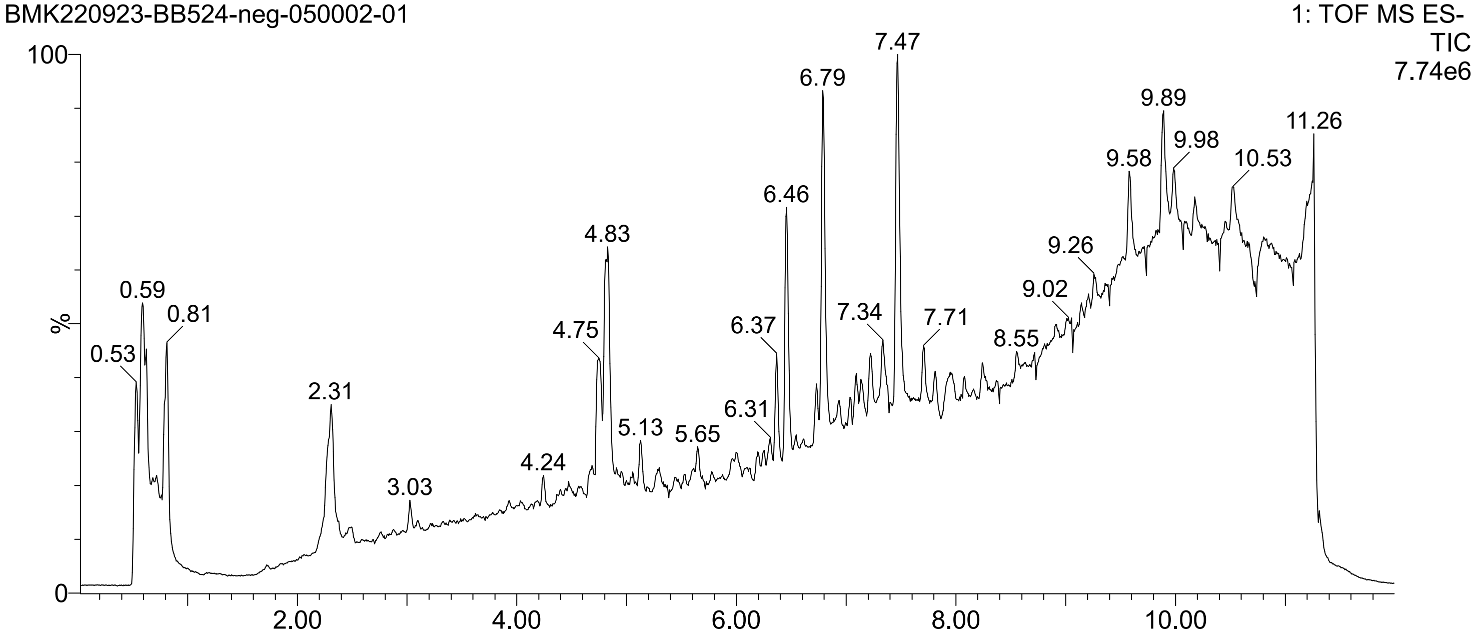
BMK220923-BB524-neg-050002-01
1: TOF MS ES-
TIC
7.74e6
100
%
0
7.47
6.79
9.89
9.98
11.26
9.58
10.53
6.46
4.83
9.26
0.59
9.02
0.81
7.34
4.75
7.71
6.37
8.55
0.53
2.31
6.31
5.13
5.65
4.24
3.03
2.00
4.00
6.00
8.00
10.00

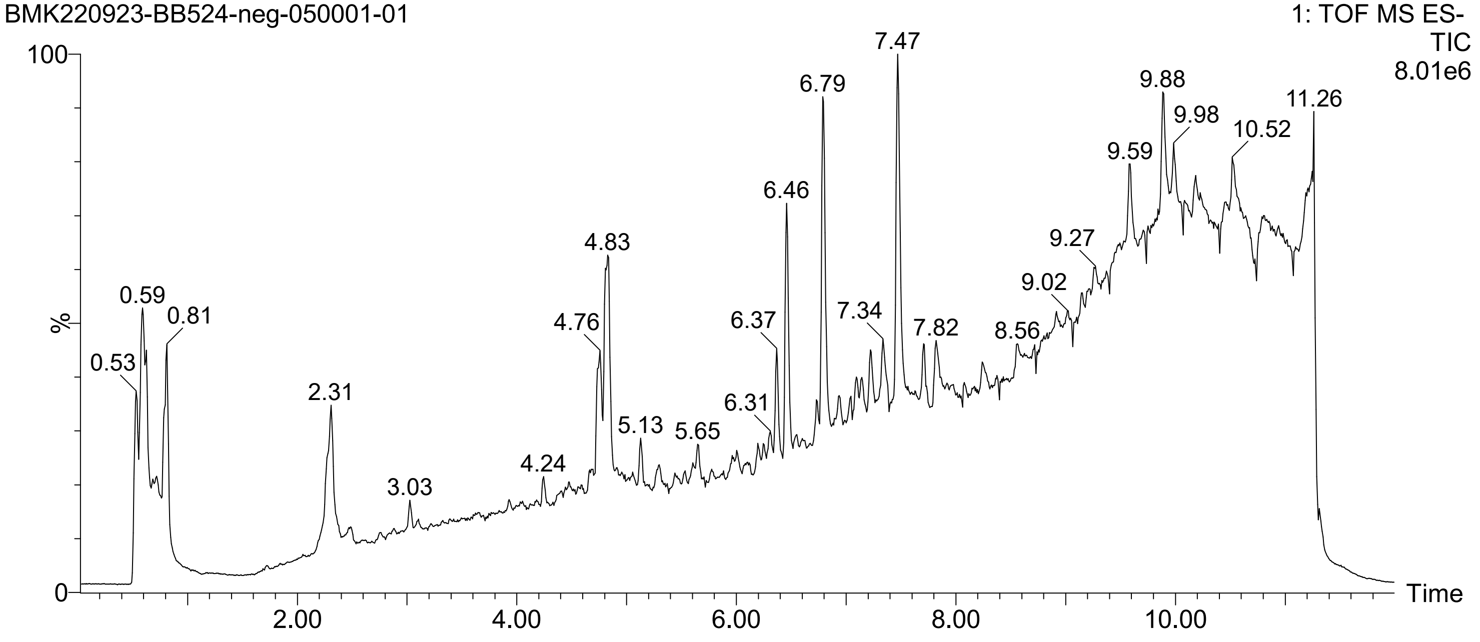
BMK220923-BB524-neg-050001-01
1: TOF MS ES-
TIC
8.01e6
100
%
0
7.47
6.79
9.88
11.26
9.98
10.52
9.59
6.46
4.83
9.27
0.59
9.02
0.81
7.34
4.76
6.37
7.82
8.56
0.53
2.31
6.31
5.13
5.65
4.24
3.03
Time
2.00
4.00
6.00
8.00
10.00

BMK220923-BB524-neg-050003-01

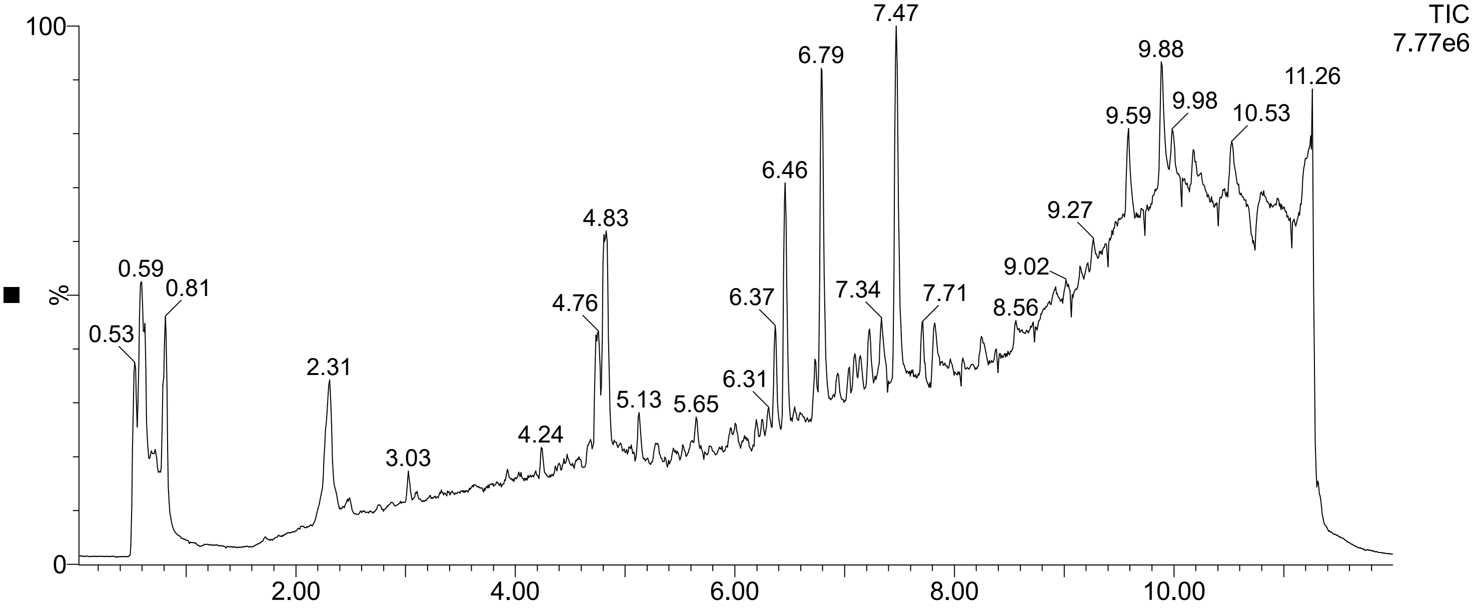

BMK220923-BB524-neg-050007-01

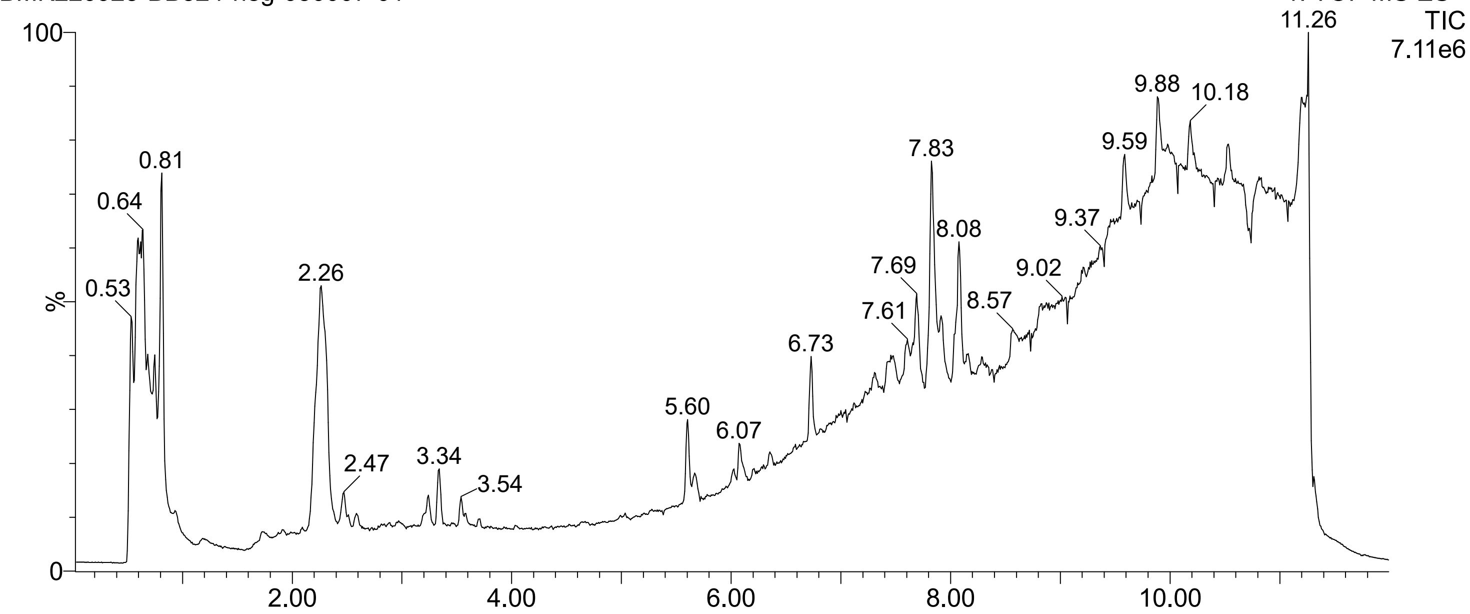

BMK220923-BB524-neg-050005-01

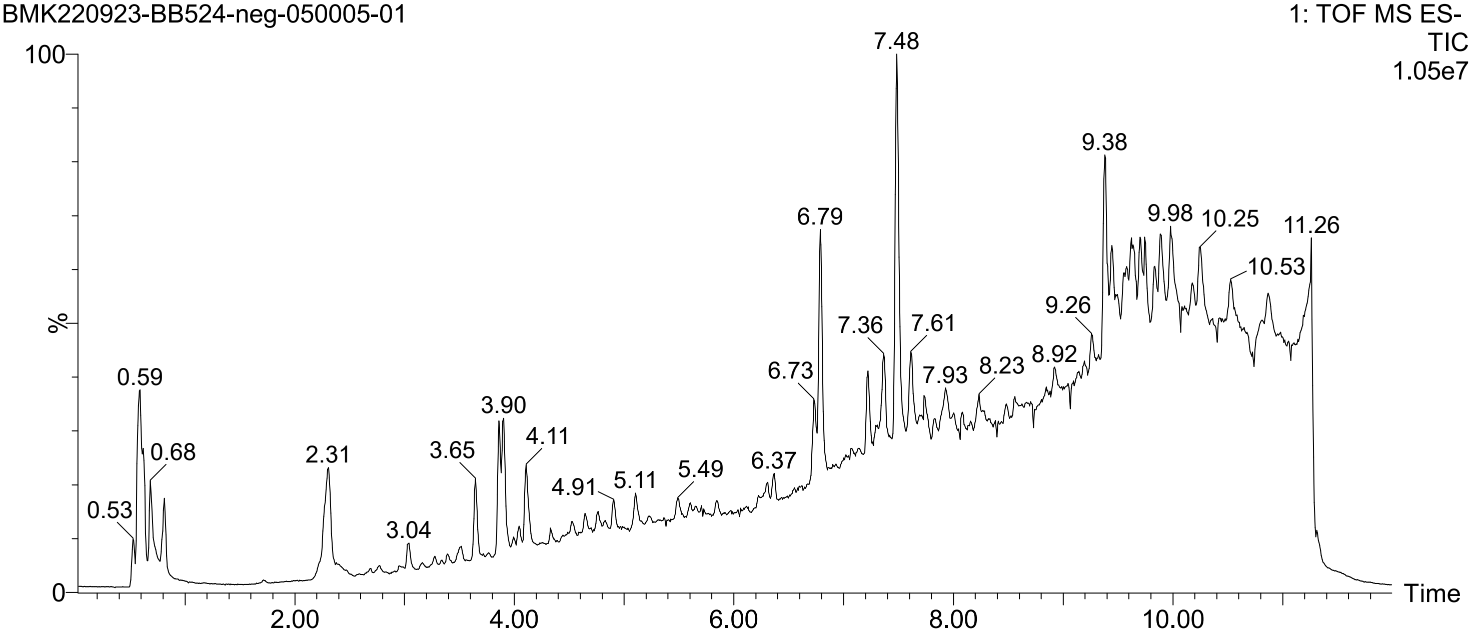

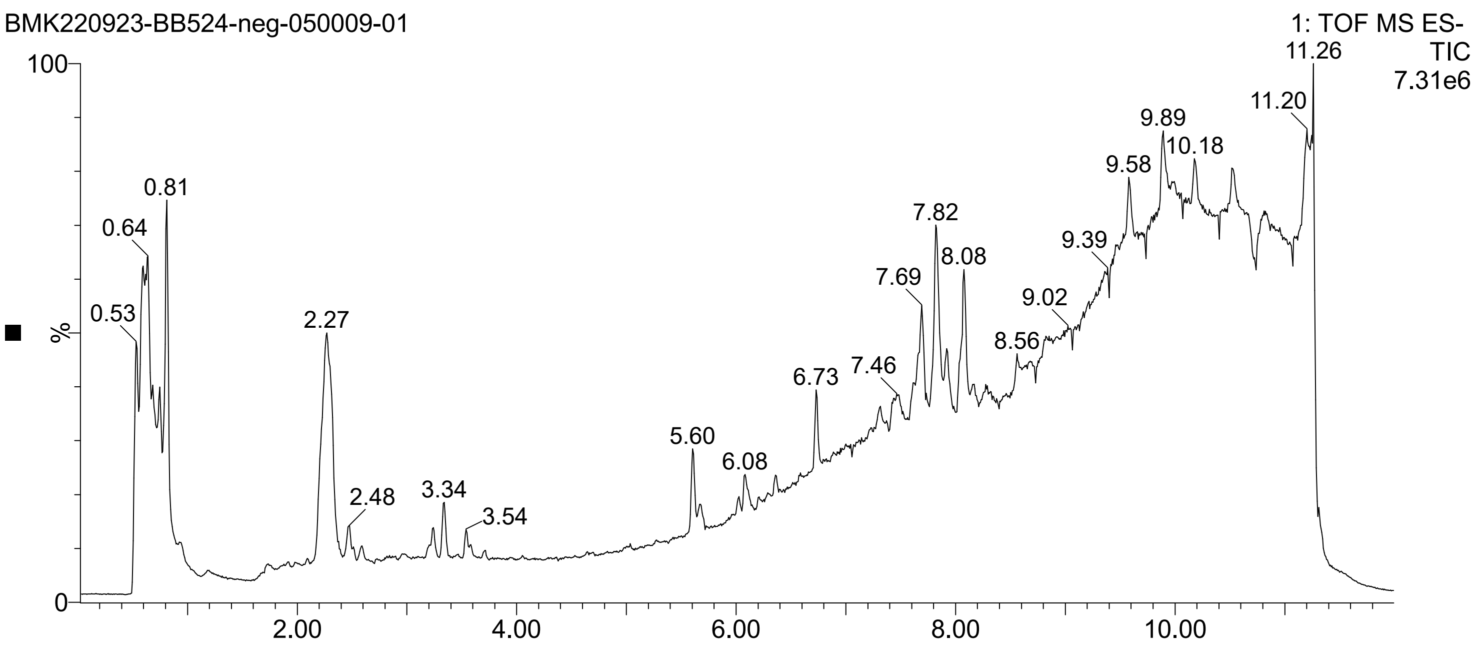

BMK220923-BB524-neg-050009-01
1: TOF MS ES-
TIC
7.31e6
100
%
0
11.26
11.20
9.89
10.18
9.58
0.81
0.64
7.82
9.39
8.08
7.69
0.53
2.27
9.02
8.56
6.73
7.46
5.60
6.08
3.34
2.48
3.54
2.00
4.00
6.00
8.00
10.00

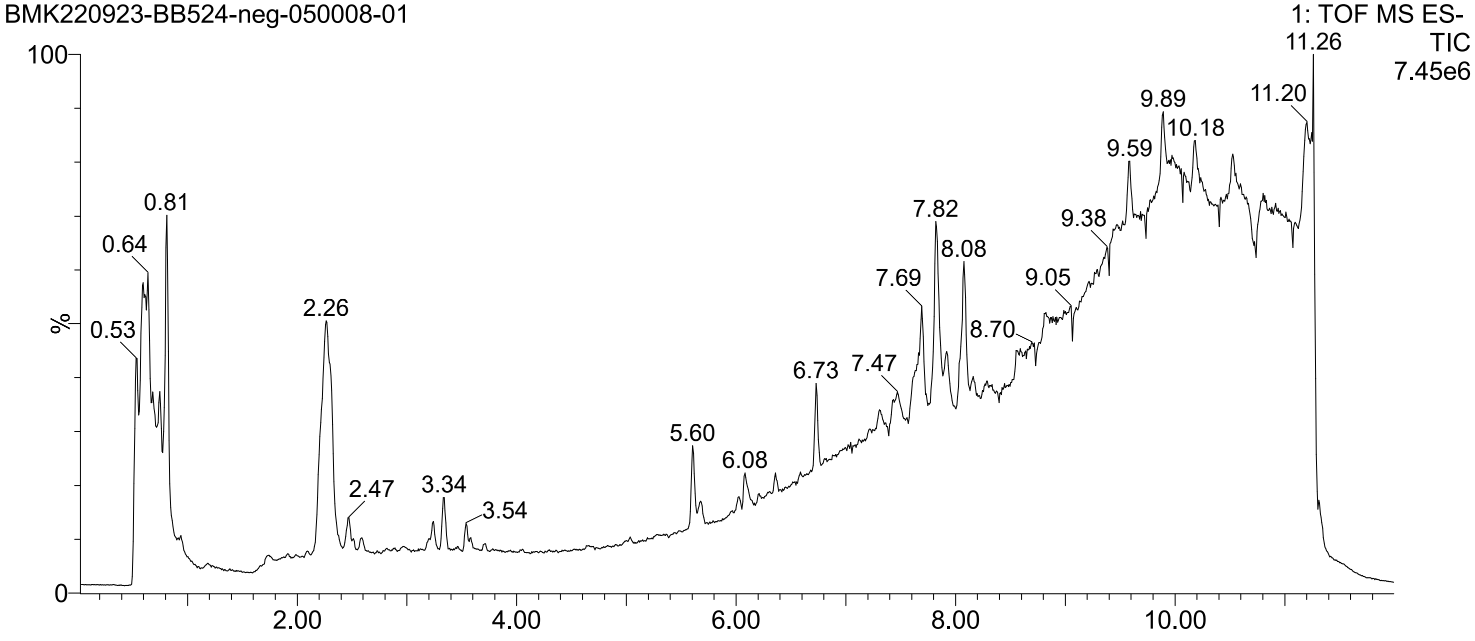

BMK220923-BB524-neg-050008-01
1: TOF MS ES-
TIC
7.45e6
100
%
0
11.26
9.89
11.20
10.18
9.59
0.81
7.82
0.64
9.38
8.08
7.69
9.05
2.26
0.53
8.70
6.73
7.47
5.60
6.08
3.34
2.47
3.54
2.00
4.00
6.00
8.00
10.00

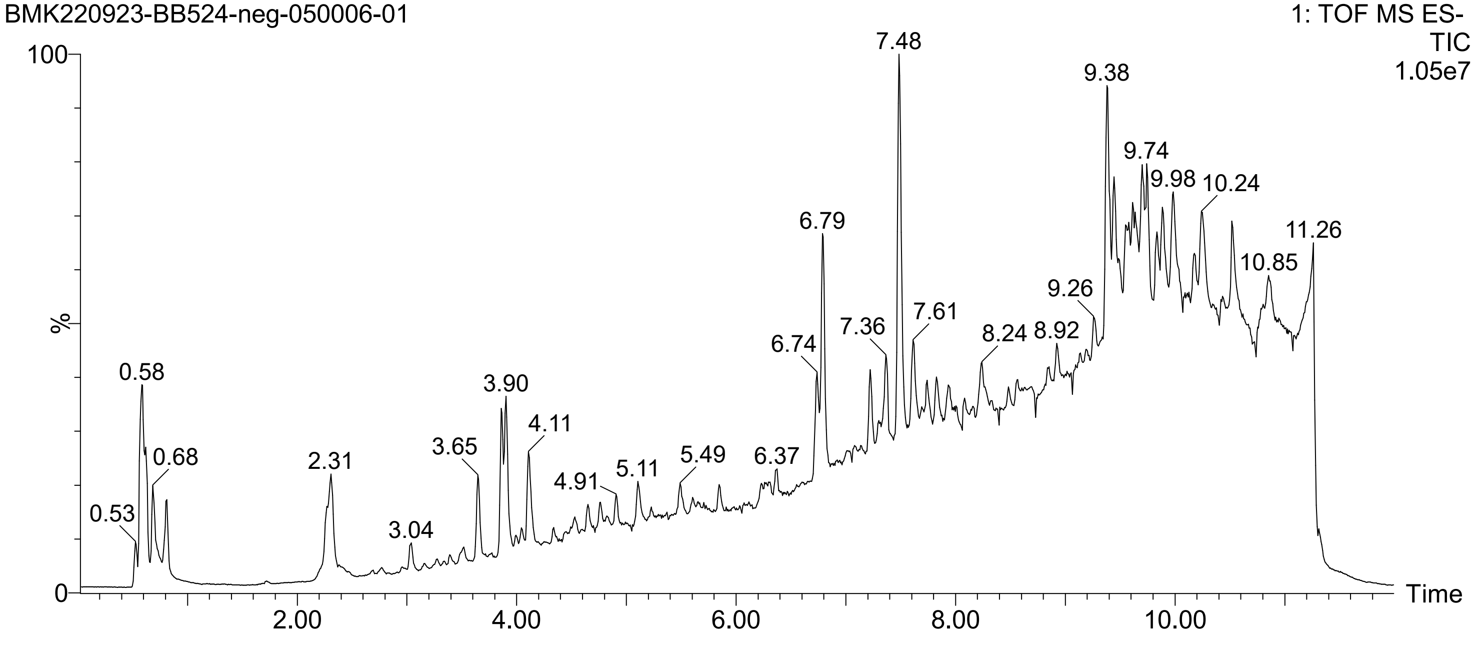

BMK220923-BB524-neg-050006-01
1: TOF MS ES-
TIC
1.05e7
100
%
0
7.48
9.38
9.74
9.98
10.24
6.79
11.26
10.85
9.26
7.36
7.61
6.74
8.24
8.92
0.58
3.90
4.11
3.65
0.68
2.31
5.49
6.37
4.91
5.11
0.53
3.04
Time
2.00
4.00
6.00
8.00
10.00

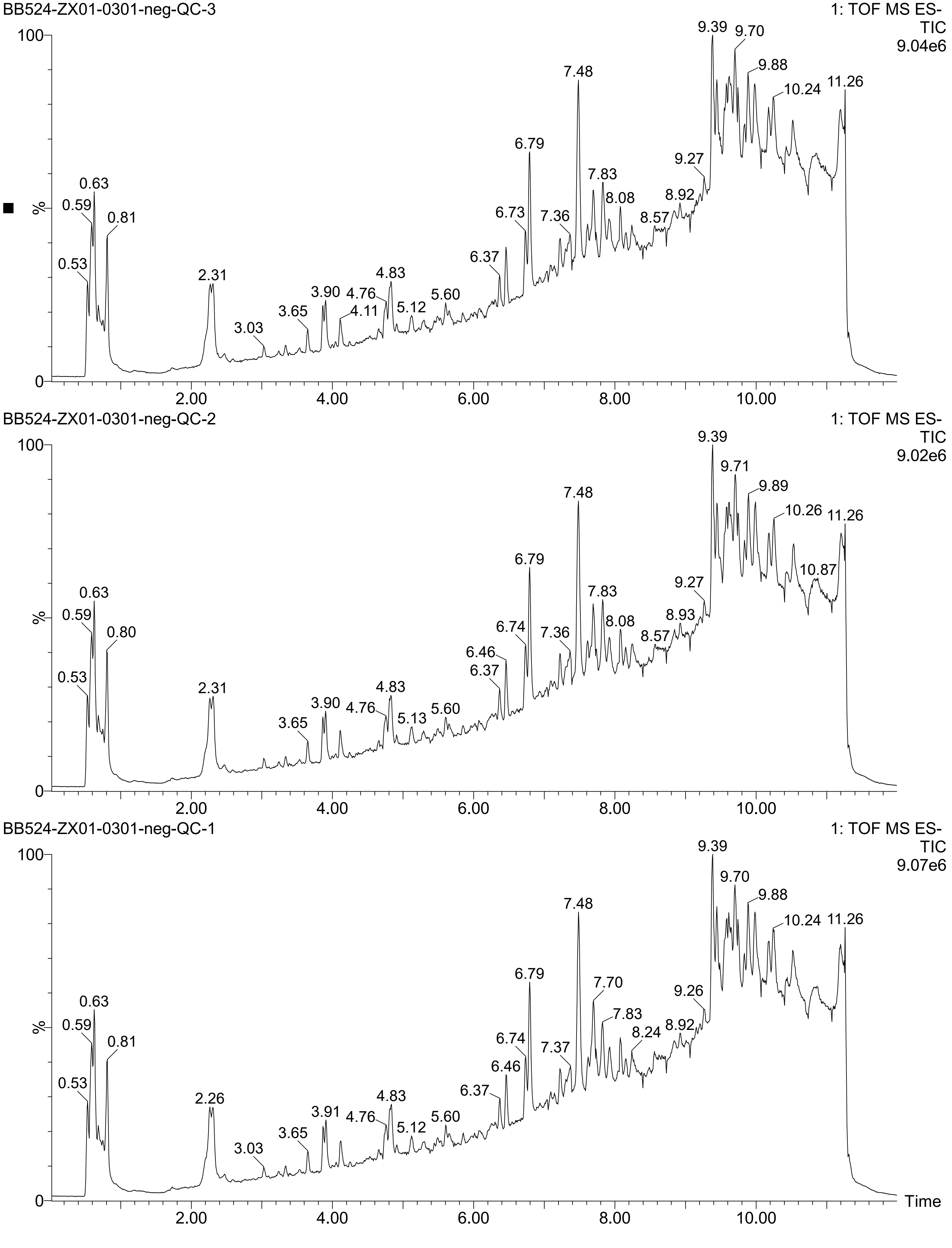
BB524-ZX01-0301-neg-QC-3
1: TOF MS ES-
TIC
9.04e6
100
%
0
0.53
0.59
0.63
0.81
2.31
3.03
3.65
3.90
4.11
4.76
4.83
5.12
5.60
6.37
6.73
6.79
7.36
7.48
7.83
8.08
8.57
8.92
9.27
9.39
9.70
9.88
10.24
11.26
2.00
4.00
6.00
8.00
10.00
BB524-ZX01-0301-neg-QC-2
1: TOF MS ES-
TIC
9.02e6
100
%
0
0.53
0.59
0.63
0.80
2.31
3.65
3.90
4.76
4.83
5.13
5.60
6.37
6.46
6.74
6.79
7.36
7.48
7.83
8.08
8.57
8.93
9.27
9.39
9.71
9.89
10.26
10.87
11.26
2.00
4.00
6.00
8.00
10.00
BB524-ZX01-0301-neg-QC-1
1: TOF MS ES-
TIC
9.07e6
100
%
0
0.53
0.59
0.63
0.81
2.26
3.03
3.65
3.91
4.76
4.83
5.12
5.60
6.37
6.46
6.74
6.79
7.37
7.48
7.70
7.83
8.24
8.92
9.26
9.39
9.70
9.88
10.24
11.26
2.00
4.00
6.00
8.00
10.00
Time
