# Supplementary material for: Multiomic Analysis Provided Insights into the Responses of Carbon Sources by Wood-Rotting Fungi Daldinia carpinicola
Source: J Fungi (Basel). 2025 Feb 4;11(2):115. doi: 10.3390/jof11020115 (PMC11856974; doi:10.3390/jof11020115)
Supplement: Supplementary file 1 [file jof-11-00115-s001.zip › jof-3369885-supplementary/Metabolome data/BMK220923-BB524-ZX01-0301.data.tar.gz!/input/pos-total.pdf]

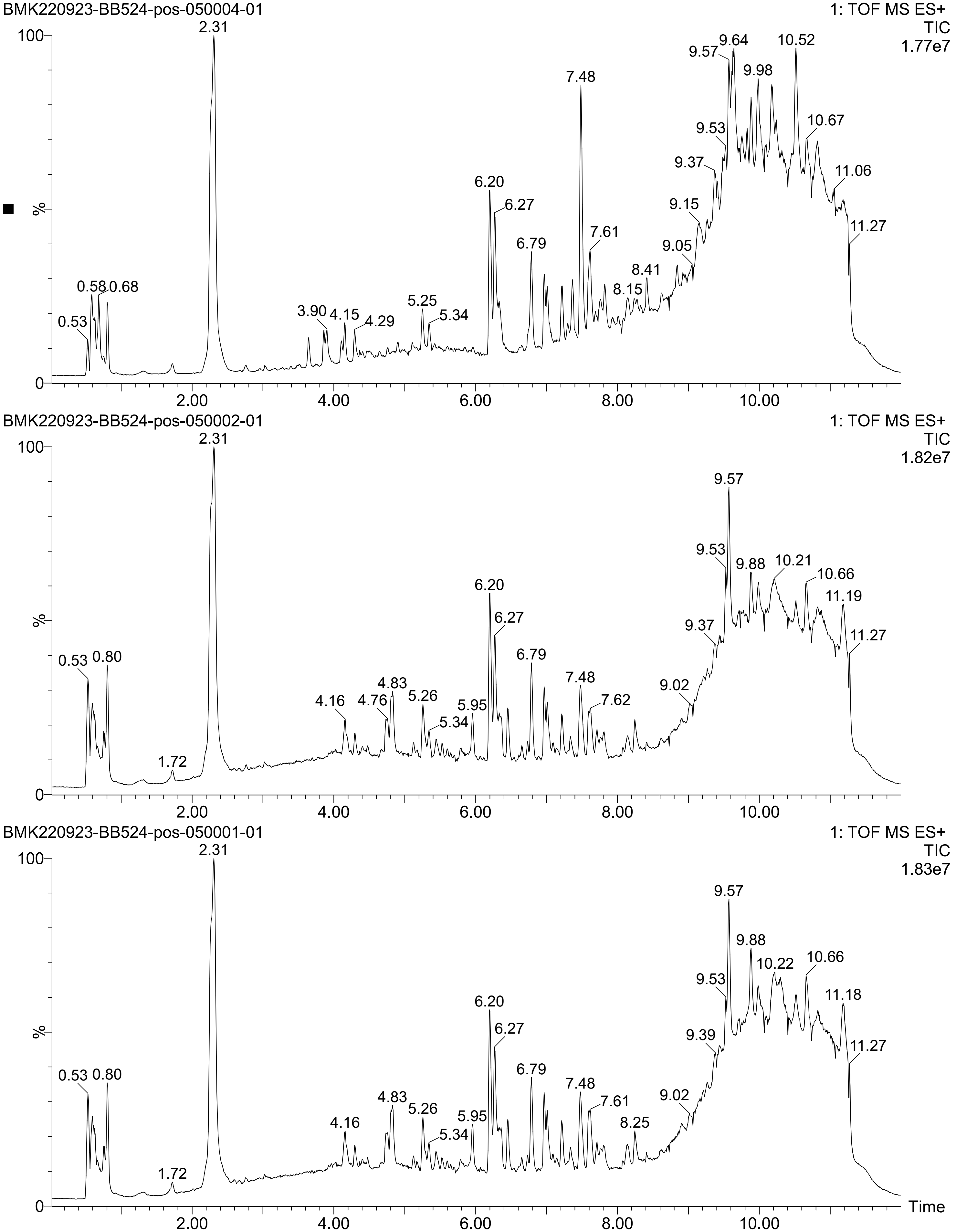

BMK220923-BB524-pos-050004-01
1: TOF MS ES+
TIC
1.77e7
2.31
0.53
0.58
0.68
3.90
4.15
4.29
5.25
5.34
6.20
6.27
6.79
7.48
7.61
8.15
8.41
9.05
9.15
9.37
9.53
9.57
9.64
9.98
10.52
10.67
11.06
11.27
BMK220923-BB524-pos-050002-01
1: TOF MS ES+
TIC
1.82e7
2.31
0.53
0.80
1.72
4.16
4.76
4.83
5.26
5.34
5.95
6.20
6.27
6.79
7.48
7.62
9.02
9.37
9.53
9.57
9.88
10.21
10.66
11.19
11.27
BMK220923-BB524-pos-050001-01
1: TOF MS ES+
TIC
1.83e7
2.31
0.53
0.80
1.72
4.16
4.83
5.26
5.34
5.95
6.20
6.27
6.79
7.48
7.61
8.25
9.02
9.39
9.53
9.57
9.88
10.22
10.66
11.18
11.27
%
100
0
2.00
4.00
6.00
8.00
10.00
Time

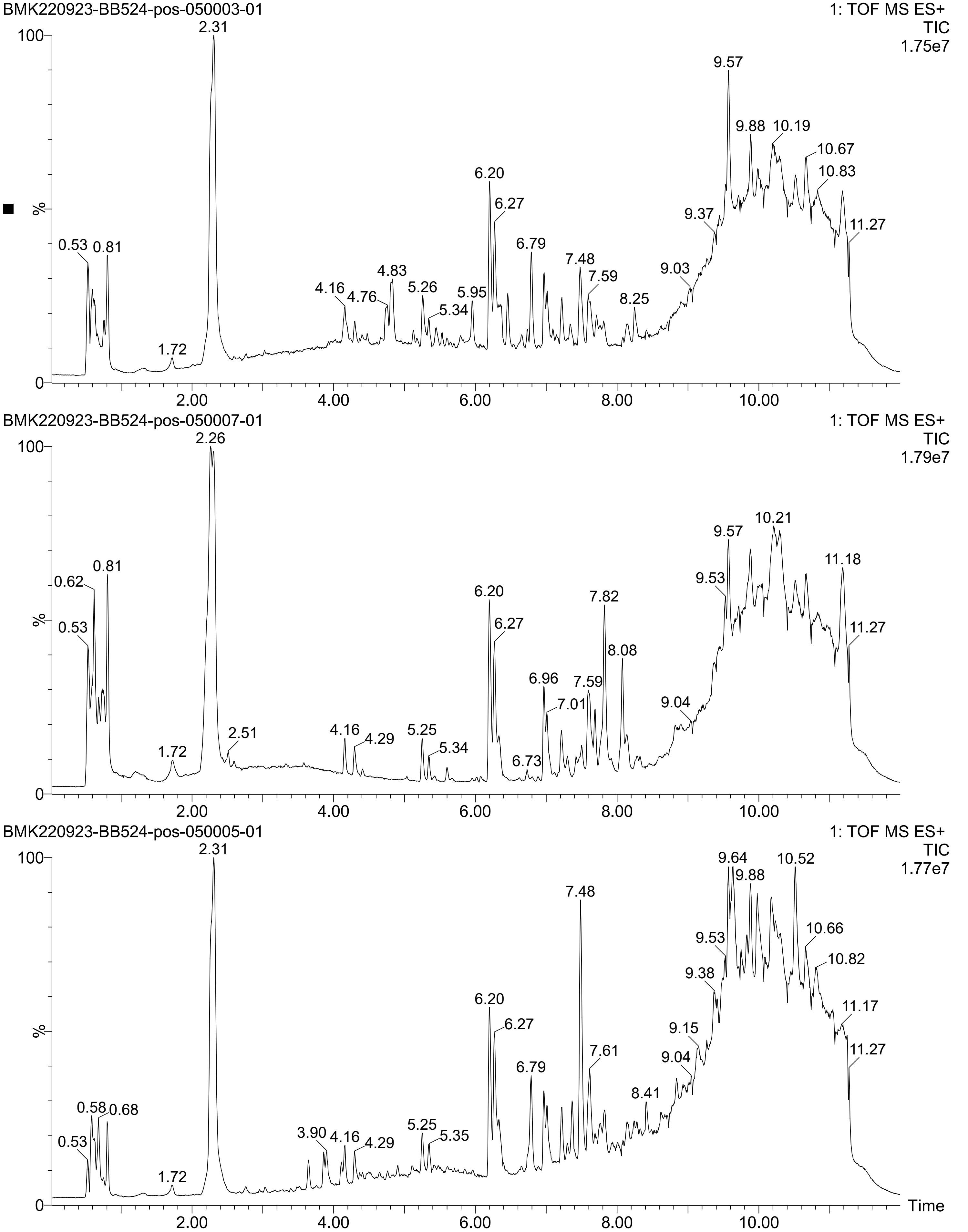
BMK220923-BB524-pos-050003-01
1: TOF MS ES+
TIC
1.75e7
100
%
0
2.31
9.57
9.88
10.19
10.67
10.83
6.20
6.27
9.37
11.27
0.53
0.81
6.79
7.48
7.59
9.03
4.83
4.16
4.76
5.26
5.95
5.34
8.25
1.72
2.00
4.00
6.00
8.00
10.00
BMK220923-BB524-pos-050007-01
1: TOF MS ES+
TIC
1.79e7
100
%
0
2.26
10.21
9.57
0.81
11.18
0.62
9.53
6.20
7.82
0.53
6.27
11.27
8.08
6.96
7.59
7.01
9.04
2.51
4.16
5.25
4.29
5.34
1.72
6.73
2.00
4.00
6.00
8.00
10.00
BMK220923-BB524-pos-050005-01
1: TOF MS ES+
TIC
1.77e7
100
%
0
2.31
9.64
9.88
10.52
7.48
10.66
9.53
10.82
9.38
6.20
11.17
6.27
9.15
7.61
11.27
6.79
9.04
8.41
0.58
0.68
3.90
4.16
5.25
5.35
0.53
4.29
1.72
Time
2.00
4.00
6.00
8.00
10.00

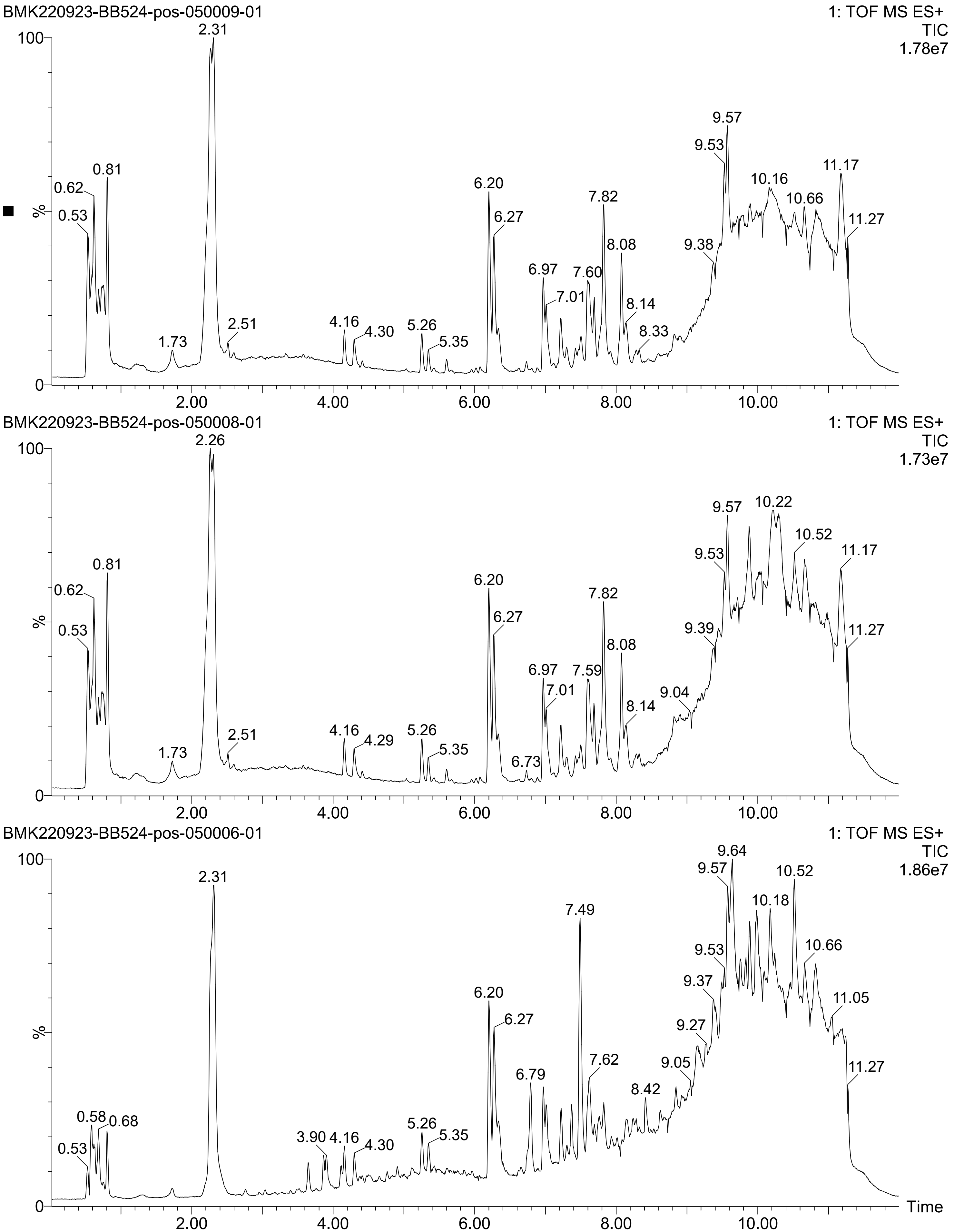

BMK220923-BB524-pos-050009-01
1: TOF MS ES+
TIC
1.78e7
100
%
0
2.31
0.81
0.62
0.53
1.73
2.51
4.16
4.30
5.26
5.35
6.20
6.27
6.97
7.01
7.60
7.82
8.08
8.14
8.33
9.38
9.53
9.57
10.16
10.66
11.17
11.27
2.00
4.00
6.00
8.00
10.00
BMK220923-BB524-pos-050008-01
1: TOF MS ES+
TIC
1.73e7
100
%
0
2.26
0.81
0.62
0.53
1.73
2.51
4.16
4.29
5.26
5.35
6.20
6.27
6.73
6.97
7.01
7.59
7.82
8.08
8.14
9.04
9.39
9.53
9.57
10.22
10.52
11.17
11.27
2.00
4.00
6.00
8.00
10.00
BMK220923-BB524-pos-050006-01
1: TOF MS ES+
TIC
1.86e7
100
%
0
2.31
0.58
0.68
0.53
3.90
4.16
4.30
5.26
5.35
6.20
6.27
6.79
7.49
7.62
8.42
9.05
9.27
9.37
9.53
9.57
9.64
10.18
10.52
10.66
11.05
11.27
2.00
4.00
6.00
8.00
10.00
Time

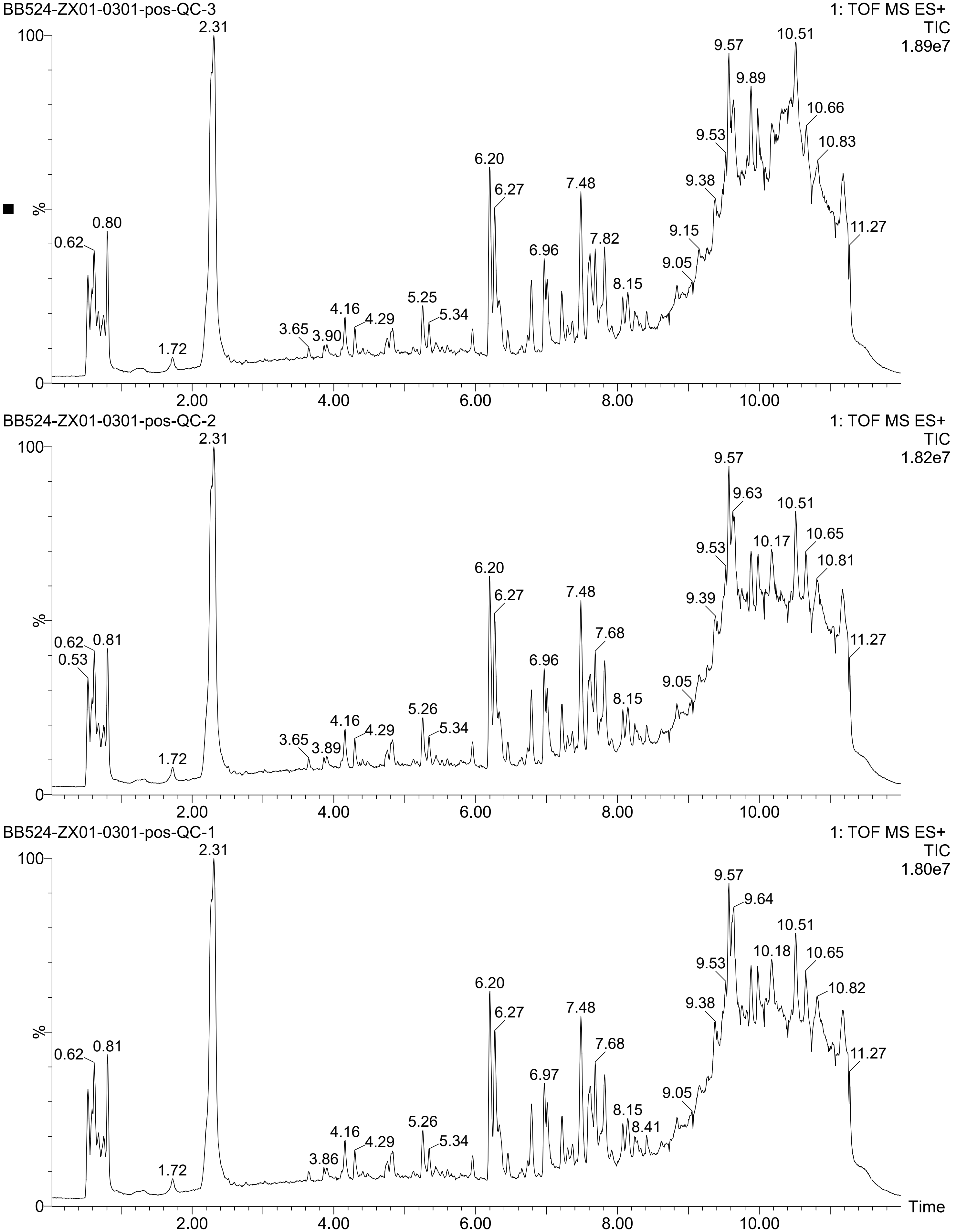
BB524-ZX01-0301-pos-QC-3
1: TOF MS ES+
TIC
1.89e7
%
2.31
0.62
0.80
1.72
3.65
3.90
4.16
4.29
5.25
5.34
6.20
6.27
6.96
7.48
7.82
8.15
9.05
9.15
9.38
9.53
9.57
9.89
10.51
10.66
10.83
11.27
BB524-ZX01-0301-pos-QC-2
1: TOF MS ES+
TIC
1.82e7
%
2.31
0.62
0.53
0.81
1.72
3.65
3.89
4.16
4.29
5.26
5.34
6.20
6.27
6.96
7.48
7.68
8.15
9.05
9.39
9.53
9.57
9.63
10.17
10.51
10.65
10.81
11.27
BB524-ZX01-0301-pos-QC-1
1: TOF MS ES+
TIC
1.80e7
%
2.31
0.62
0.81
1.72
3.86
4.16
4.29
5.26
5.34
6.20
6.27
6.97
7.48
7.68
8.15
8.41
9.05
9.38
9.53
9.57
9.64
10.18
10.51
10.65
10.82
11.27
100
0
2.00
4.00
6.00
8.00
10.00
Time
