# Supplementary material for: Multiomic Analysis Provided Insights into the Responses of Carbon Sources by Wood-Rotting Fungi Daldinia carpinicola
Source: J Fungi (Basel). 2025 Feb 4;11(2):115. doi: 10.3390/jof11020115 (PMC11856974; doi:10.3390/jof11020115)
Supplement: Supplementary file 1 [file jof-11-00115-s001.zip › jof-3369885-supplementary/Transcriptome KEGG map/ko01040.html]

ko01040
